# Fluorescence imaging of bombesin and transferrin receptor expression is comparable to ^18^F-FDG PET in early detection of sorafenib-induced changes in tumor metabolism

**DOI:** 10.1371/journal.pone.0182689

**Published:** 2017-08-08

**Authors:** Jen-Chieh Tseng, Nara Narayanan, Guojie Ho, Kevin Groves, Jeannine Delaney, Bagna Bao, Jun Zhang, Jeffrey Morin, Sylvie Kossodo, Milind Rajopadhye, Jeffrey D. Peterson

**Affiliations:** Discovery & Analytical Solutions R&D, PerkinElmer Inc., Hopkinton, Massachusetts, United States of America; Biomedical Research Foundation, UNITED STATES

## Abstract

Physical measurement of tumor volume reduction is the most commonly used approach to assess tumor progression and treatment efficacy in mouse tumor models. However, it is relatively insensitive, and often requires long treatment courses to achieve gross physical tumor destruction. As alternatives, several non-invasive imaging methods such as bioluminescence imaging (BLI), fluorescence imaging (FLI) and positron emission tomography (PET) have been developed for more accurate measurement. As tumors have elevated glucose metabolism, ^18^F-fludeoxyglucose (^18^F-FDG) has become a sensitive PET imaging tracer for cancer detection, diagnosis, and efficacy assessment by measuring alterations in glucose metabolism. In particular, the ability of ^18^F-FDG imaging to detect drug-induced effects on tumor metabolism at a very early phase has dramatically improved the speed of decision-making regarding treatment efficacy. Here we demonstrated an approach with FLI that offers not only comparable performance to PET imaging, but also provides additional benefits, including ease of use, imaging throughput, probe stability, and the potential for multiplex imaging. In this report, we used sorafenib, a tyrosine kinase inhibitor clinically approved for cancer therapy, for treatment of a mouse tumor xenograft model. The drug is known to block several key signaling pathways involved in tumor metabolism. We first identified an appropriate sorafenib dose, 40 mg/kg (daily on days 0–4 and 7–10), that retained ultimate therapeutic efficacy yet provided a 2–3 day window post-treatment for imaging early, subtle metabolic changes prior to gross tumor regression. We then used ^18^F-FDG PET as the gold standard for assessing the effects of sorafenib treatment on tumor metabolism and compared this to results obtained by measurement of tumor size, tumor BLI, and tumor FLI changes. PET imaging showed ~55–60% inhibition of tumor uptake of ^18^F-FDG as early as days 2 and 3 post-treatment, without noticeable changes in tumor size. For comparison, two FLI probes, BombesinRSense^™^ 680 (BRS-680) and Transferrin-Vivo^™^ 750 (TfV-750), were assessed for their potential in metabolic imaging. Metabolically active cancer cells are known to have elevated bombesin and transferrin receptor levels on the surface. In excellent agreement with PET imaging, the BRS-680 imaging showed 40% and 79% inhibition on days 2 and 3, respectively, and the TfV-750 imaging showed 65% inhibition on day 3. In both cases, no significant reduction in tumor volume or BLI signal was observed during the first 3 days of treatment. These results suggest that metabolic FLI has potential preclinical application as an additional method for detecting drug-induced metabolic changes in tumors.

## Introduction

Current preclinical evaluation of anti-cancer treatments using animal models relies predominantly on physical measurements of the reduction of tumor volume. This is a relatively insensitive measure of anti-tumor effects, requiring repeated drug dosing and frequent measurements, often over multiple weeks. Furthermore, this approach provides little or no information directly regarding drug mechanism or early treatment response. For a treatment to be effective, the drug should induce biological changes at the cellular level prior to overt physical changes in tumor size as a whole. Thus the ability to detect these subtle yet significant biological changes can be very informative in early drug development, particularly when the candidate drug molecules may not have dramatic therapeutic effects at the dose level tested. One of the early biological changes in tumors is altered energy metabolism. In the 1920’s, Otto Warburg discovered that tumors preferably use glycolysis as the energy production pathway in order to survive in an anaerobic environment [1, 2]. This phenomenon, named the Warburg effect, was observed in many types of human cancer. Researchers and clinicians since the late 1980’s [3] took advantage of the Warburg effect and developed a glucose analog ^18^F-fludeoxyglucose (^18^F-FDG) for PET imaging. Since then, this imaging technology has become the gold standard imaging method for both tumor detection and efficacy assessment of anti-cancer therapies in clinic [4–7]. As energy production plays a critical role in cancer proliferation and survival, it is not surprising that several key oncogenic signaling regulators directly control cellular metabolic status. For example, the Ras/Raf/MEK/ERK signaling is known to cross-talk with the Akt/mTOR pathways that directly promote glucose metabolism [8]. Therefore, targeting these important regulatory molecules should cause immediate metabolic decrease in tumor cells prior to eventual tumor volume regression, a complex and slow process involving both tumor cells and normal host cells.

Although ^18^F-FDG PET imaging is ideal for efficacy assessment of targeted therapy, widespread adoption of PET imaging in preclinical research laboratories can be limited by access to PET imagers, availability of radionuclide-labeled probes, and the extra precautions and safety guidelines required. As an alternative, there are many non-radioactive, FLI tools currently available for pre-clinical readouts, offering benefits of ready access, safety, probe stability, and the potential for multiplexing with more than one imaging probe. Indeed, many have shown that fluorescent imaging probes can be used to investigate a variety of important biological activities in tumor, such as vascular leak [9], hypoxia [10, 11], integrin expression [12], cell death [13], and protease activity [14]. For metabolism imaging, some researchers have even explored the utility of near-infrared (NIR) fluorescent glucose probes for non-invasive imaging of glucose metabolism in tumors [15, 16] as a potential optical equivalent of ^18^F-FDG. However, size restriction of the ligand-binding pocket on glucose transporters prevents uptake of NIR-labeled glucose [17], since fluorescence modification significantly increases the size of glucose. We took an alternative approach to assessing tumor metabolism and developed two NIR fluorescent probes, BombesinRSense 680 (BRS-680) and Transferrin-Vivo 750 (TfV-750), for tumor metabolic imaging. These two probes allow non-invasive imaging of bombesin and transferrin receptor expression levels on tumor cells as indicators of active energy metabolism [18, 19].

Bombesin is a 14-amino acid peptide originally isolated from the European fire-belly toad (*Bombina bombina*) [20]. Bombesin–like peptides and bombesin receptors (BBRs) have been the subject of investigation for the past two decades because of their involvement in cancer cell energy metabolism and proliferation. Mammalian bombesin analogs, such as gastrin-releasing peptide (GRP), can promote cell growth [21], malignant transformation, and tumor differentiation [22], so it is not surprising that many types of human cancer, including prostate, breast, lung, CNS, gastric, colon, and renal, show upregulated expression of BBRs [23]. Taking advantage of broad BBR tumor expression, radiolabeled bombesin peptide analogs have been used effectively as PET imaging tracers in cancer research [24, 25]. Based on the same principle, fluorescent BRS-680 was developed as an optical alternative for preclinical imaging of cancer, using a 7-amino acid sequence from GRP that was labeled with a NIR fluorophore (ex/em 674/690 nm) and conjugated with a pharmacokinetic modifier to improve its bioavailability and circulation half-life (plasma t_1/2_ ~ 1.7h).

In addition to BRS-680, TfV-750 is the other imaging probe available for visualizing tumor energy metabolism. We developed TfV-750 as a NIR FLI probe specific for cell surface transferrin receptors by using PEGylated recombinant transferrin and a NIR fluorescent reporter (ex/em 750/770 nm). The receptors’ ligand, transferrin is a 79-kDa monomeric plasma glycoprotein that binds and transports iron to all tissues through receptor-mediated endocytosis [26]. This transport is critical for the cell to maintain its cellular biochemistry, metabolism and proliferation. In particular, tumor cells have stringent demand for iron and overexpress transferrin receptors in order to support their rapid cell metabolism and growth, making the transferrin receptor a useful tumor imaging biomarker [27].

We hypothesized that FLI of bombesin and transferrin receptor expression can be used for early detection of treatment-induced tumor metabolic changes in a manner similar to the gold standard of ^18^F-FDG PET imaging [28]. To test this, we quantified BRS-680 and TfV-750 longitudinal imaging of HCT116-luc2 xenograft tumors in treated and untreated nude mice. In order to induce metabolic shock in tumors, we used sorafenib, a clinically approved tyrosine protein kinase inhibitor for the treatment of primary liver cancer and thyroid carcinoma [29, 30]. The drug is a small molecule inhibitor of several tyrosine kinases, including PDGFR, VEGFR and Raf kinases [31]. As Raf plays a key role in the Ras/Raf/MEK/ERK pathway and is involved in regulating energy metabolism in cancer cells [8], this inhibition adversely affects tumor metabolic activity. For validation, we performed multiplex optical imaging of BLI, BRS-680, and TfV-750 and compared the results with those obtained by ^18^F-FDG PET imaging. Our data demonstrated the robustness of FLI as a valid tumor imaging method for visualizing early metabolic responses to anti-cancer treatment.

## Materials and methods

### Cell lines and reagents

The HCT116-luc2 cell line is one of PerkinElmer’s Bioware Ultra^™^ Cell lines (Waltham, MA). This human colon cancer cell line was originally derived from parental HCT116 cells (CCL-247, ATCC), and we used a pGL4 luc2 lentiviral vector to stably express firefly luciferase for BLI. The cells were maintained in McCoy 5A medium supplemented with 10% fetal bovine serum. Sorafenib (p-toluenesulfonate salt) was purchased from the LC Laboratories (Woburn, MA). BombesinRSense 680 and Transferrin-Vivo 750 FLI probes are products of PerkinElmer Inc.

### Animal tumor model and treatments

Specific pathogen-free nu/nu (Crl:NU-*Fox1*^*nu*^, 6–8 weeks old) and CD-1 (Crl:CD1[ICR]) mice were purchased from Charles River Laboratories (Wilmington, MA). Mice were housed in a controlled environment (72°F; 12:12-h light-dark cycle) under specific-pathogen free conditions with water and food provided *ad libitum*. To induce tumor growth, 2.5 x10^6^ of HCT116-luc2 cells were subcutaneously injected on the right flank of a nude mouse. Two weeks after cancer cell inoculation, when the tumor volume reached an appropriate size (measured by caliper and calculated by the formula 0.5 x length x width^2^), animals were randomized into vehicle or different sorafenib treatment groups as indicated. Sorafenib was prepared in water containing 6.2% of ethanol and 6.2% of Kolliphor EL (Sigma-Aldrich, St. Louis, MO). The treatment was given daily 5 times a week by oral gavage at 15 μl per gram of body weight.

### Fluorescent imaging probes

The two commercially available NIR FLI probes used in these studies, BRS-680 and TfV-750, were developed, characterized, and validated by PerkinElmer R&D. The molecular weight of BRS-680 is approximately 24,000 g/mol, with an excitation/emission profile determined as 674/694 nm (S1 Fig). Selectivity was assessed *in vitro* using HT-29 human colorectal cells; fluorescence signal on BRS-680-labeled cells was ~7X greater than either the scrambled peptide version of the probe or when 100X unlabeled bombesin peptide was used to block labeling (S2 Fig) as assessed by both flow cytometry and fluorescence microscopy. The plasma pharmacokinetic profile was assessed in CD-1 mice with sequential bleeds measured in a fluorescence microplate assay to determine the circulation half-life as ~1.7h. Collected tissues from HT-29 tumor-bearing nu/nu mice showed good biodistribution to tumors, predominant kidney clearance, and the expected binding to receptors in the pancreas (S3 Fig). *In vivo* tumor imaging was optimal at 24h (S4 Fig) with near complete clearance by 7–8 days (data not shown). Tumor tissue localization co-localized well with *ex vivo* anti-bombesin receptor staining (S5 Fig).

The molecular weight of TfV-750 is approximately 106,000 g/mol, with an excitation/emission profile determined as 750/770 nm (S6 Fig). Selectivity was assessed in vitro by flow cytometry and fluorescence microscopy using HT-29 human colorectal cells; fluorescence signal on TfV-750-labeled cells was ~15X greater than when 100 μM unlabeled recombinant transferrin was used to block labeling (S7 Fig). Plasma pharmacokinetics of TfV-750 was assessed in CD-1 mice. Animals were bled at indicated times, and plasma fluorescence was determined using a microplate assay. The circulation half-life of TfV-750 is ~9h, and FLI imaging of collected tissues from HT-29 tumor-bearing nu/nu mice indicates good biodistribution to tumors, efficient clearance via kidney excretion, and expected uptake in the liver (S8 Fig). The optimal timeframe for *in vivo* tumor imaging is from 6 to 24h post injection, and time required for complete clearance from the system is about 4 days (S9 Fig). The probe has good tissue penetration into tumors, as its binding pattern in tumor sections (using a 645 nm FLI surrogate to improve microscopy imaging) correlates well with the transferrin receptor expression on cancer cells and is not associated with the tumor vasculature (S10 Fig).

### Molecular imaging procedures

Optical BLI and FLI were performed using the IVIS^®^ SpectrumCT, which is capable of imaging multiple mice simultaneously (up to 5 mice). To generate BLI signals, the tumor-bearing mice received IP injections of 75 mg/kg of D-luciferin (potassium salt, PerkinElmer Inc.) and were placed into the imager for sequential image acquisitions (settings: FOV = D, Binning = 16, F/Stop = 1, Open filter, 2 min delay between each acquisition) under 1–2% of isoflurane anesthesia. Maximal bioluminescence output for each tumor was determined by sequentially acquiring a BLI image every two minutes for up to 16 min. Typical peak imaging time for the HCT116-luc2 subcutaneous model was approximately 10 min, and BLI images are represented in units of radiance (p/sec/cm^2^/sr).

In addition, the imager is equipped with suitable excitation and emission filter sets for FLI of BRS-680 and TfV-750. Tumor metabolic activities were visualized by IV injection of a mixture of these two NIR imaging agents (2 nmol/mouse each in a total volume of 100 μl of PBS). The agents were injected 16 h prior to FLI imaging in order to allow optimal probe uptake and clearance of unbound probe. For the longitudinal FLI imaging studies, separate cohorts were used for FLI acquisitions on days 1 (*n* = 10, imaging agents injected on day 0), 2 (*n* = 10, injected on day 1), and 3 (*n* = 5, injected on day 2) due to the extended time required for probe washout. In particular, part of the day 1 cohort (*n* = 5) was re-injected with probe on day 9 for imaging on day 10 to assess ultimate treatment efficacy. To acquire BRS-680 fluorescent images, the mice were placed in the imaging chamber and imaged with the following settings: FOV = C, Binning = 2, F/Stop = 2, Ex 640/Em 680, shutter time = 2 sec. TfV-750 images were acquired with similar settings except for the use of an Ex 750/ Em 800 filter set. Fluorescent signal is represented as radiant efficiency [(p/sec/cm^2^/sr)/(μW/cm^2^)] or normalized fluorescent signal where appropriate.

We also performed microPET imaging using ^18^F-FDG as a tracer for detecting changes in tumor glucose uptake with or without treatment. Images were acquired using the G8 PET/CT system (PerkinElmer Inc.). For each mouse, 100 μCi of ^18^F-FDG (PETNET Solutions Inc., Malvern PA) was injected intravenously (IV), and all mice were kept warm and maintained under 1.5% isoflurane gas anesthesia for 1 hour for tracer uptake. Subsequently, the mice were imaged with a standard 10 min static F-18 imaging protocol. The G8 PET/CT system automatically performed image reconstruction after acquisition and provided 3D tomography imaging in the DICOM format. We were able to image the same cohorts (*n* = 5) throughout the course of study since ^18^F has a relative short decay half-life (~110 min). We used the VivoQuant 3.0 software (inviCRO Inc., Boston MA) for PET data viewing and analysis. Tumor specific glycolytic activity is represented as SUV_total_ or normalized uptake where appropriate.

### Data and statistical analysis

BLI and FLI images were analyzed using the Living Image software (version 4.4 for Windows, PerkinElmer Inc.). Tumor BLI and FLI signals were quantified using the region of interest (ROI) tools, and the total optical signals were calculated as total flux (photon counts/sec) and total radiant efficiency [(p/s)/(μW/cm^2^)], respectively. For greater accuracy, all FLI data was corrected for background levels based on specific measurements as described in the figure legends. All PET images were represented as standard uptake value (SUV) using the VivoQuant 3.0 software and tumor-specific ^18^F-FDG uptake levels were also calculated using SUV_total_. Several similar imaging experiments were performed using this particular HCT116-luc2 tumor model to visualize treatment responses to sorafenib. Power analysis of these preliminary datasets suggested a sample size of 5–10 is satisfactory (α = 0.05, β = 0.1) for this type of imaging study. For each treatment group, we calculated relative tracer uptake in tumors using the day 0 uptake level as 100%. For statistical analysis, we used the GraphPad Prism software (version 6.0 for Windows, La Jolla, CA). Data are presented as the mean ± standard error of the mean (s.e.m.). Statistical analysis was conducted using ANOVA or Student’s *t*-test, as indicated. *P* < 0.05 was the cutoff for being considered statistically significant.

### Histology

Tumor tissues were fixed in 10% neutral buffered formalin, followed by routine process and embedment in paraffin. Tissue sections (5 μm) were prepared on electrostatically charged glass slides and then baked at 60°C overnight. After washing the slides with three washes in xylene, the slides were rehydrated through a series of graded ethanol (100%, 90% and 70%) to water. The prepared slides were then stained with hematoxylin and eosin.

### Ethics statement

All experiments were performed in accordance with the recommendations in the Guide for the Care and Use of Laboratory Animals of the National Institutes of Health, and an ARRIVE Guidelines check list for this study is included (S11 Fig). The protocol (#01–0904) was approved by PerkinElmer’s (*In Vivo* Imaging Division) IACUC guidelines for animal care and use. No invasive or surgical procedures were used in these studies, but all imaging activities were performed under appropriate anesthesia to minimize animal distress.

## Results

### Molecular imaging of tumor regression in response to anti-cancer treatment

**Fig 1:**
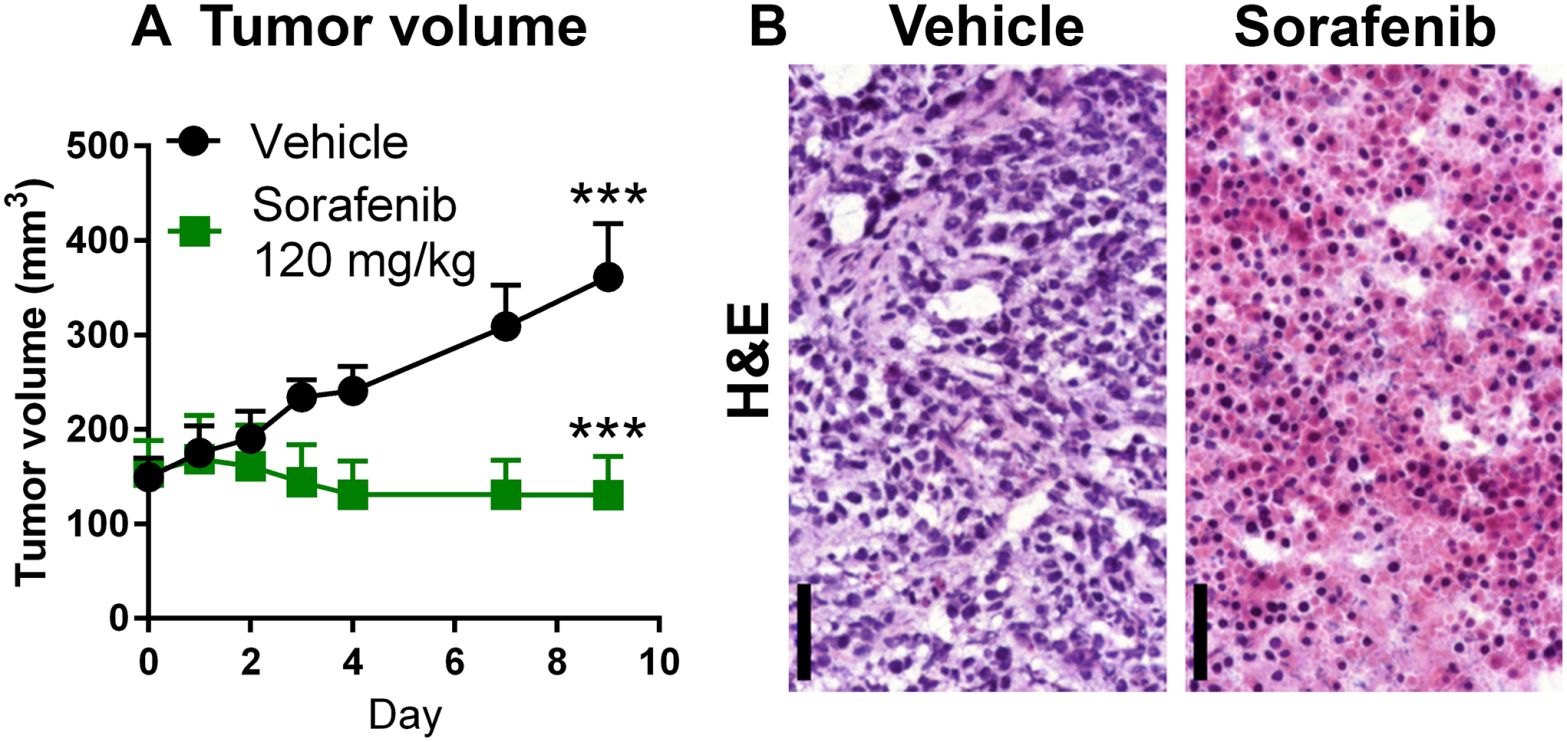
Sorafenib, a tyrosine kinase inhibitor, effectively suppresses HCT116-luc2 tumor growth. Nude mice bearing subcutaneous tumors on the flanks were treated with vehicle or with 120 mg/kg of sorafenib on days 0–4 and days 7–9 via oral gavage. **A:** Longitudinal tumor volume measurements of subcutaneous HCT116-luc2 tumors (***p < 0.0001, *n* = 5 per group, two-way ANOVA, mean and s.e.m., representative of three studies) **B:** H&E staining of tumor tissue sections harvested from the vehicle and sorafenib-treated (120 mg/kg) tumors on day 9. The drug effectively induced necrosis in treated tumors. Bar: 50 μm.

To test the imaging strategy for early metabolic changes in tumors, we first established a tumor xenograft treatment model that responds well to a Ras/Raf/MEK/ERK targeted therapy. In this study, we used nu/nu mice bearing human HCT116-luc2 subcutaneous xenografts implanted on the flank and imaged their responses to the sorafenib treatment. Sorafenib is a small molecular inhibitor of several tyrosine kinases, including PDGFR, VEGFR and Raf kinases [31]. We initiated the treatment when the average HCT116-luc2 tumor size for each group reached ~180 mm^3^ in volume (day 0). The drug was given daily via oral gavage at a dosage of 120 mg/kg from days 0 to 4 and from days 7 to 9. In comparison to vehicle-treated animals, conventional tumor volume measurements by caliper clearly demonstrated the drug’s efficacy against HCT116-luc2 tumor growth (Fig 1A). Furthermore, at this dose level, sorafenib is capable of causing extensive tumor death by day 9 as visualized by microscopy of H&E stained tumor tissue sections (Fig 1B).

### Using BLI tumor viability imaging to determine a dose of sorafenib that minimizes early effects on tumor viability and size while retaining significant late efficacy

**Fig 2:**
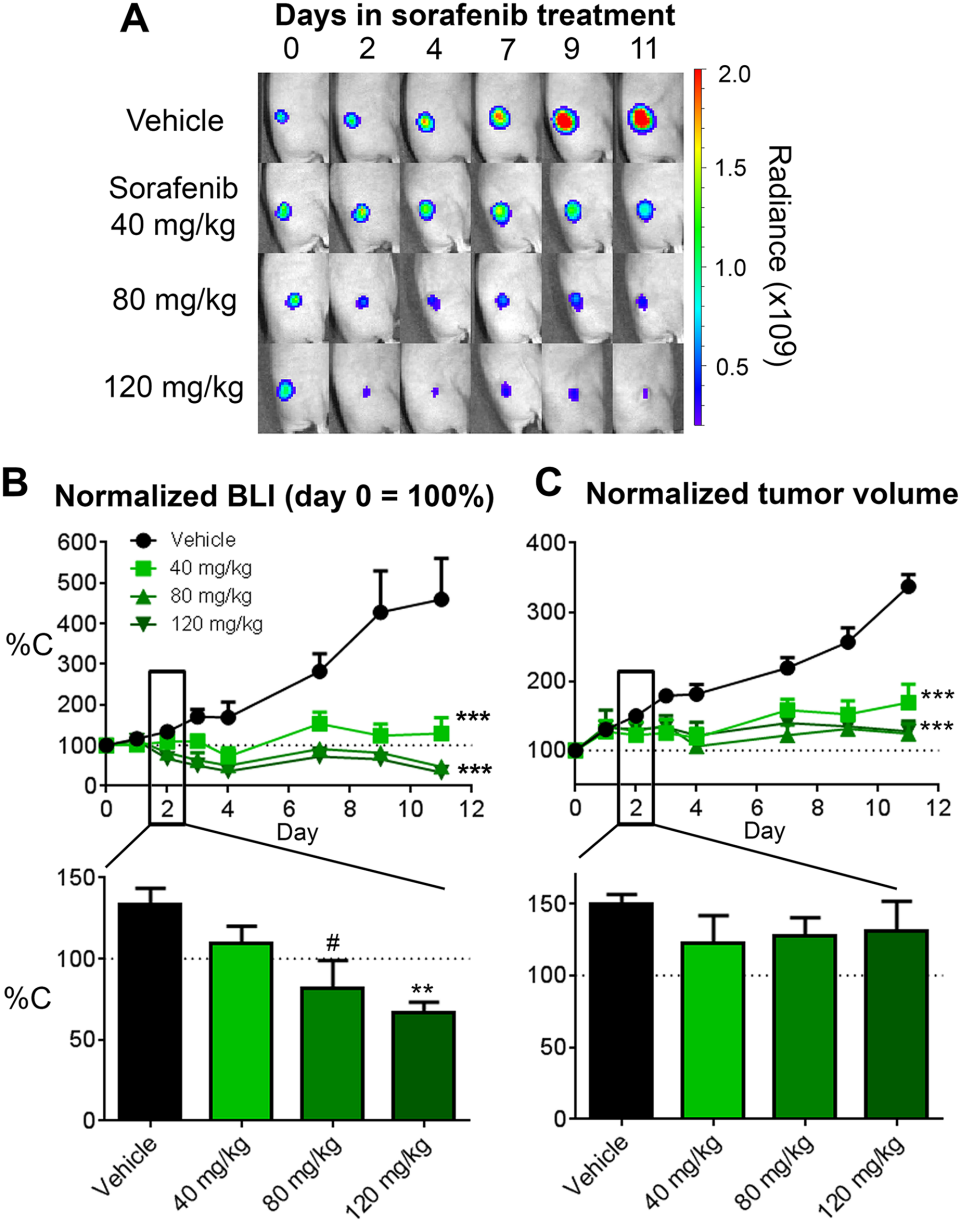
Sorafenib dose-titration study on HCT116-luc tumors in nude mice. Tumor progression was assessed by BLI and tumor volume from the start of sorafenib treatments (mice enrolled with ~50–75 mm^3^ tumor volumes) though day 11. **A:** Representative mouse images of longitudinal BLI of HCT116-luc2 tumors in response to daily treatments of 120, 80 or 40 mg/kg. Treatments were given daily on day 0–4 and day 7–11. **B:** Relative tumor BLI of firefly luciferase activity was determined for each treatment group and represented using each group’s day 0 signal as 100%. The lower panel shows relative BLI signal of each dose group on day 2. **C:** Relative tumor volumes of each treatment group calculated using the day 0 volume as 100%. The lower panel shows relative tumor volume of each dose group on day 2. Statistics for longitudinal readouts were done by two-way ANOVA (***p < 0.0001, *n* = 5 per group, representative of three studies) with data represented as mean and s.e.m. The bar charts in the lower panels show the mean relative tumor readouts on day 2 with statistics assessed by Student’s t-test (^#^*p*<0.05, n = 5 per group).

Since sorafenib targets the Ras/Raf/MEK/ERK pathway which is also involved in regulating energy metabolism in cancer cells [8], we hypothesized that treatment could reduce tumor metabolism and viability almost immediately upon initiation of treatment. There should be an early window of time in which sorafenib treatment alters tumor energy metabolism prior to cell death and overt changes in tumor size. The optimal scenario for assessing biological changes would be to separate tumor regression from the precipitating biological changes by decreasing the treatment dose to prolong the time window prior to onset of tumor regression. In that regard, we performed a sorafenib dose-titration study to identify the minimal effective dose of sorafenib that leads to effective long-term tumor regression but has little overt effect on tumor size or viability during the first couple of days of treatment. In this set of studies (Fig 2), treatments were initiated when the average group HCT116-luc2 tumor size reached ~100 mm^3^ in volume. Three sorafenib dose levels (120, 80 and 40 mg/kg), all within the published efficacious dose range of the drug, were tested on nu/nu mice bearing subcutaneous luciferase-expressing HCT116-luc2 xenografts on the flank. The drug was given daily, from days 0 to 4 and from days 7 to 11. In addition to the conventional tumor volume measurements by caliper, we performed BLI of firefly luciferase activity to access tumor viability on the days indicated. Bioluminescence signal generally correlated well with cell viability status as light generation by the luciferase enzyme requires ATP. The BLI imaging results revealed a good correlation between treatment dose and the degree of tumor growth suppression (Fig 2A). Quantitative representation of the imaging data indicated that, at the highest dose of 120 mg/kg, sorafenib effectively suppressed HCT116-luc2 tumor viability (~90%) in living animals by day 11 (Fig 2B), a finding consistent with the tumor volume measurement (Fig 2C). In addition, at this high dose, substantial reduction in tumor BLI signals can be observed as early as 2 days (~40%) after the treatment began, whereas tumor volume showed no significant change (Fig 2C), suggesting its effects on tumor viability even at this early time. Comparable results were seen at the 80 mg/kg dose. Interestingly, animals treated with 40 mg/kg sorafenib showed no significant reduction in size (tumor volume) or viability (assessed by BLI) within 2 days of treatment. Nevertheless, this lower dose retained sufficient efficacy to suppress tumor growth at the later stage, and over multiple studies we typically observed ~50–90% growth inhibition in comparison with untreated control tumors by day 10 or 11. The benefit of the lower sorafenib dose was the broader window of time, in the first 2–3 days, in which we could measure tumor biological changes without the impact of gross tumor regression.

**Fig 3:**
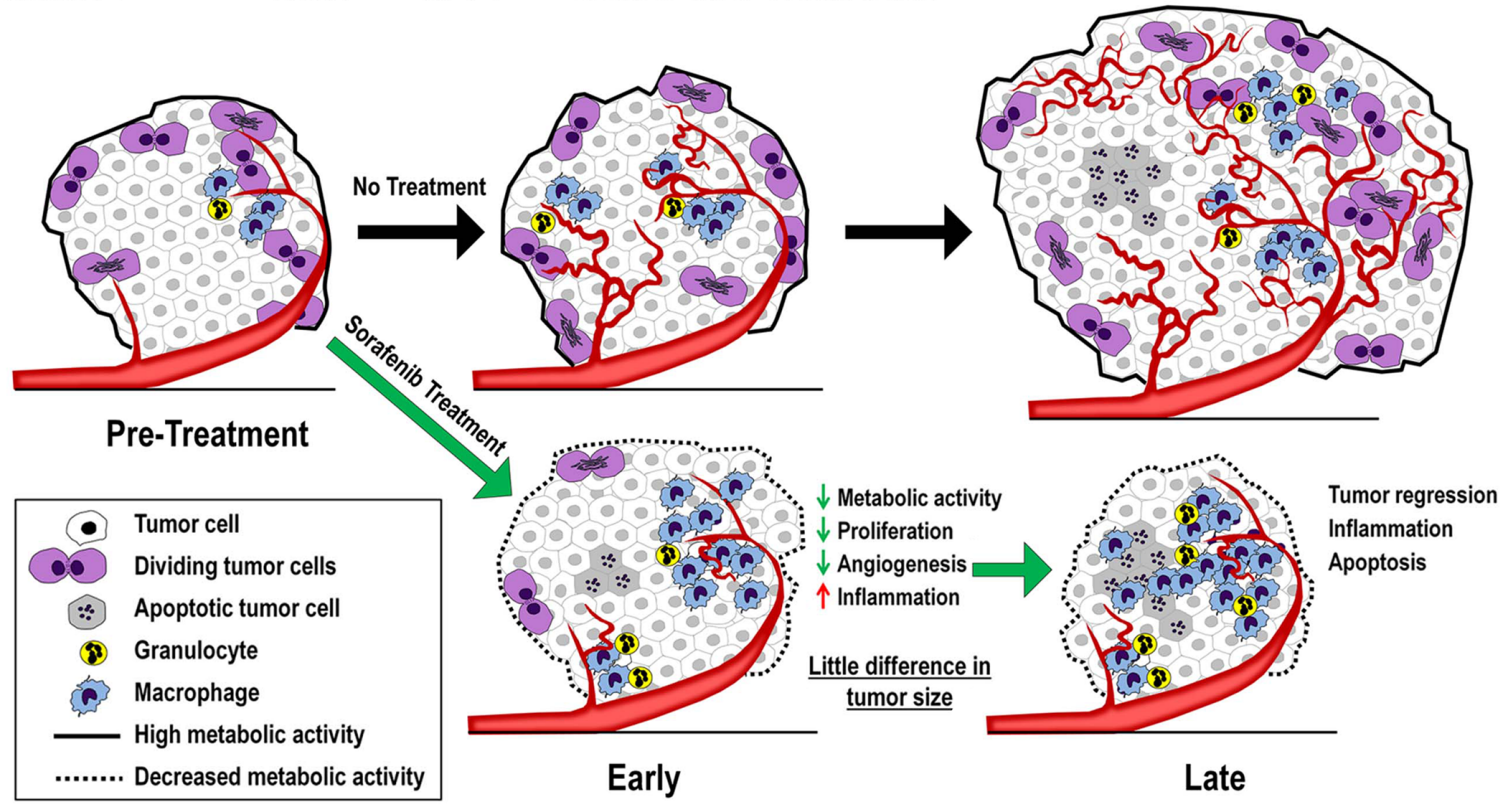
Tumor biology changes in progression and during anti-cancer treatment. There are several tumor biological changes during disease progression and in response to a targeted anti-cancer drug. Without treatment, the cancer cells are metabolic active and can produce sufficient energy for cancer cell proliferation and overall tumor growth. Active cancer cell proliferation also triggers new vasculature formation in tumor (angiogenesis) that is critical for tumor growth. On the other hand, a treatment using targeted anti-cancer drugs, such as sorafenib, can suppress metabolic activity in tumors during the immediate, early phase of treatment. Such metabolic suppression could reduce the growth of not only the cancer cells, but also the vascular endothelial cells that are responsible for angiogenesis. At the late state of treatment, the drug induces extensive tumor death which triggers inflammatory responses and recruitment of granulocytes and macrophages. We hypothesize that the transition from early to late responses can be modulated by the dose level of the targeted drug.

Obviously, since 40 mg/kg sorafenib has an effect on tumor volume by 11 days, we expected biological changes would occur in the tumor even during the first 2–3 days after the initiation treatment. During this early phase of sorafenib treatment, the drug could immediately target and inhibit tumor cell metabolic and proliferative pathways, simultaneously targeting vascular endothelial cells and angiogenesis critical for oxygen and nutrient supply for the tumor [32] and enhancing anti-tumor inflammation [33, 34]. Repeated drug exposure should lead to cumulative tumor effects, ultimately impacting tumor progression. This concept is illustrated in Fig 3. From a metabolic perspective, both the magnitude of anti-tumor effect as well as the time it takes to transition from early pathway effects to later overt tumor regression could be modulated by the dose level of the anti-cancer drug. At a high drug dose, the metabolic transition may be quick and will correlate completely with tumor viability, as the drug rapidly shuts down the cancer cell and causes its death. At a lower minimal effective dose of sorafenib, tumors could have a prolonged transition phase as the drug slowly disrupts tumor metabolism without immediately affecting viability.

### Using microPET imaging to profile tumor glucose metabolism in response to minimal effective dose of sorafenib

**Fig 4:**
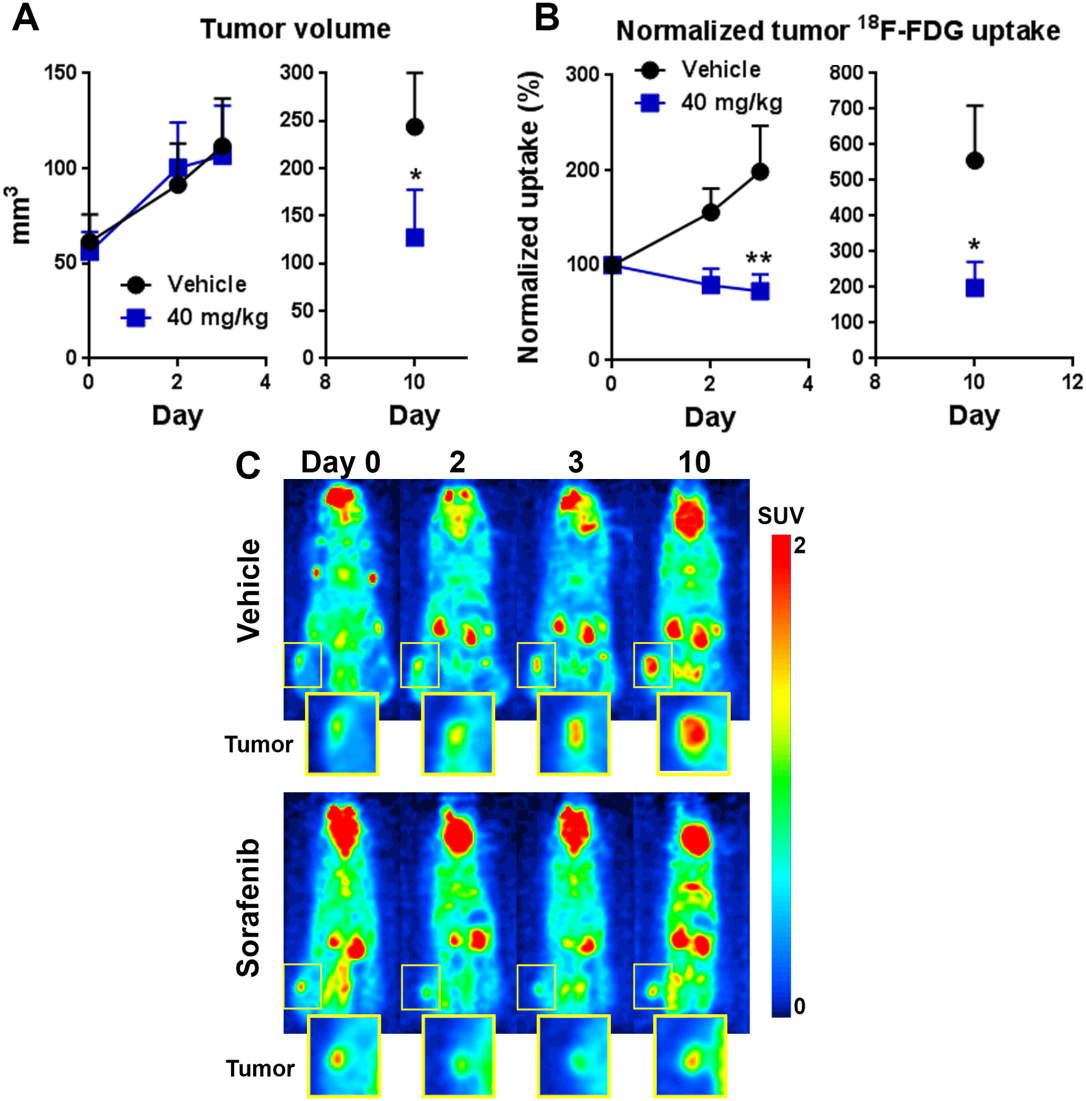
Using ^18^F-FDG PET to visualize early treatment responsiveness. **A:** Tumor volume data of mice receiving vehicle or daily sorafenib treatment at 40 mg/kg. The treatment was initiated on day 0, with mice enrolled into the study with tumor volumes ~ 50–75 mm^3^. **B:** Relative tumor ^18^F-FDG uptake levels using the day 0 data as 100%. Each animal was injected IV with 100 μCi of ^18^F-FDG and were acquired using the G8 PET/CT system after 1 h incubation period for tracer uptake (*n* = 5 per group; *p < 0.01,**p < 0.001; two-way ANOVA, mean and s.e.m., representative of two experiments). **C:** Representative microPET images of animals treated with vehicle or 40 mg/kg of sorafenib (unit: SUV_total_).

One of the key metabolic markers of tumors is the preference for using glucose as an energy source, and many types of human cancer show elevated glycolysis activity. For example, oncogenic Ras/Raf/MEK/ERK signaling is known to cross-talk with the Akt/mTOR pathways that directly promote glucose metabolism. Taking advantage of this unique feature, radioactive ^18^F-fludeoxyglucose (FDG) has been widely used as the gold standard for molecular imaging of many types of human cancer. Specific tumor uptake of ^18^F-FDG in the body can be detected non-invasively by PET, and this approach has been useful for both clinical and research applications. We used a small, high-sensitivity microPET system (G8, PerkinElmer, Waltham, MA) to perform longitudinal ^18^F-FDG microPET imaging of sorafenib (40 mg/kg) effects on HCT116-luc2 tumor metabolism (Fig 4). At all imaging time points, we also documented tumor volume changes. Similar to our previous findings, there was no significant difference during the first 2–3 days of treatment at this dose level (Fig 2), although prolonged treatment did decrease overt tumor size ~50% by day 10 (Fig 4A). Interestingly, PET imaging data indeed showed significant glucose uptake reduction as early as day 2–3 post-treatment (Fig 4B and 4C). Thus, the data shows that PET imaging can detect early suppression of glycolytic activity in the tumor prior to overt changes in tumor size/viability, consistent with previous reports using similar treatments [5, 6].

### Molecular imaging of cancer treatment outcome using multiplex BLI and FLI

**Fig 5:**
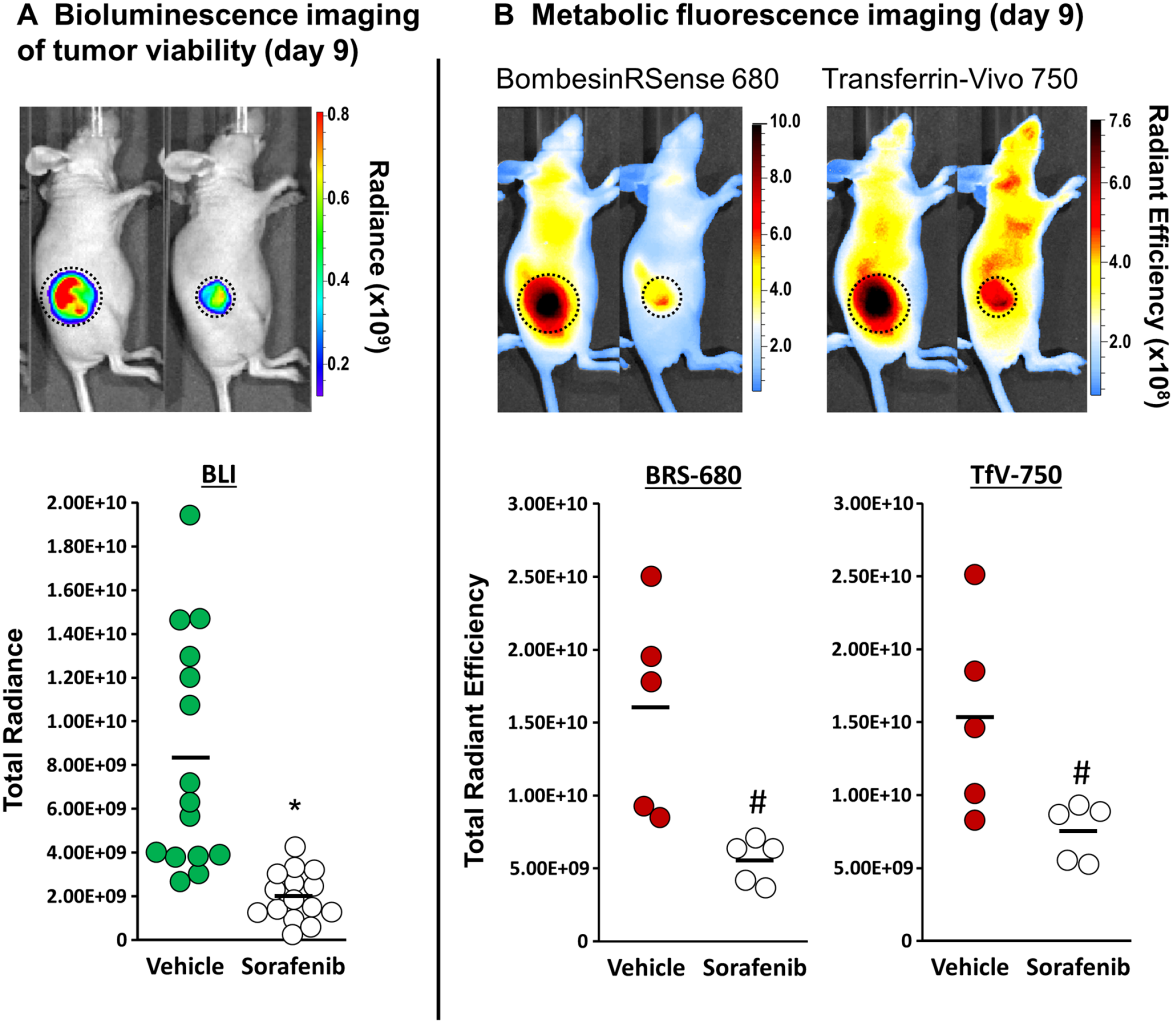
Molecular imaging of tumor viability and metabolism in response to sorafenib treatment. **A:** Images for representative mice bearing HCT116-luc2 tumors are shown that were imaged on day 9 by BLI to assess tumor burden/viability. Compared with vehicle control, HCT116-luc2 tumors treated with 120 mg/kg of sorafenib show significant quantitative reduction of BLI signal on day 9, indicating loss of tumor viability. (*p < 0.01, *n* = 15, student’s *t*-test, bar: s.e.m., representative of three independent studies) **B:** Images of tumor-bearing mice imaged using fluorescent BRS-680 and TfV-750. Both probes were administered IV on day 8 and the animals were imaged on day 9, allowing sufficient time to generate a tumor-specific fluorescent contrast. Compared with control tumors, the sorafenib treatment significantly reduced tumor metabolic activity on day 9. (^#^*p* < 0.05, *n* = 5, Student’s *t*-test, bar: s.e.m., representative of three independent studies).

We then tested a multiplex optical imaging strategy in the HCT116-luc2 tumor xenograft model (Fig 5), combining BLI with FLI of two commercially available imaging probes, BRS-680 and TfV-750 (performance and validation summarized in supplementary figures). As the majority of published optical imaging studies focus on late-stage, terminal time points, we felt it was important to first benchmark these probes’ capability for late-stage treatment assessment. To achieve this, tumor-bearing mice were treated daily either with our vehicle or with our highest sorafenib dose (120 mg/kg) for 8 days. Both FLI probes were then systemically injected IV and their specific uptake was imaged the next day. Since the unbound probes require at least 12–16 h for clearance from the tissue; this imaging strategy ensures optimal visualization of selective receptor-mediated uptake and retention in tumor cells. For multiplex imaging, as these two probes have different optical properties with little or no crosstalk (680 nm vs 750 nm), their specific uptake in tumors can be separately imaged using different optical filters. In addition, we noticed that both fluorescent agents were capable of specific tumor detection and resulted in excellent tumor definition with a sensitivity of detection of as little as 1.35-fold over background. As far as treatment efficacy and detection sensitivity, we are able to see up to 40% or more reduction in signal as compared to controls. Furthermore, we then assessed tumor viability by performing BLI imaging of the tumors. To do so, tumor-bearing mice were injected IP with luciferin for BLI acquisition 10–15 minutes later. Performing BLI after FLI imaging prevents any possible crosstalk of BLI signal that could potentially be detected in 680 nm FLI acquisitions and thus facilitated high quality triple acquisition of BLI and 2 FLI probes’ uptake in tumors on the same day. As expected, we found that tumors from sorafenib-treated animals had significantly lower bioluminescence, BRS-680, and TfV-750 signal as tumors regressed at this later stage of treatment (Fig 5). This means that metabolic BRS-680 and TfV-750 tumor signal can both substitute for BLI in instances in which researchers cannot use luciferase-expressing tumor cell lines. A variety of other FLI probes can also show such utility in imaging late stage treatment outcomes [9–14].

### Detecting early sorafenib-induced tumor metabolic changes by FLI

**Fig 6:**
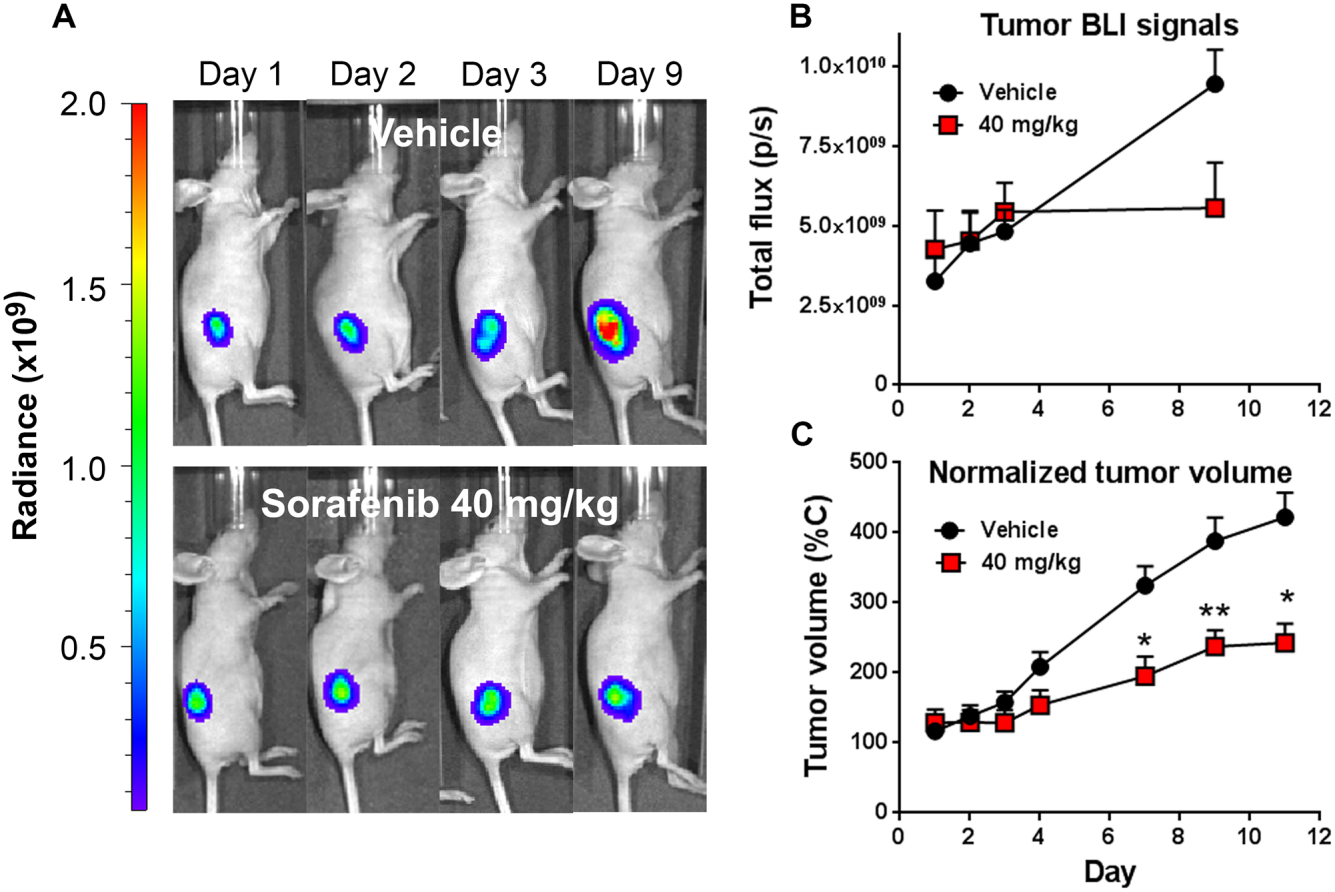
Monitor changes of HCT116-luc2 tumor viability in response to low dose sorafenib. Viability of HCT116-luc2 tumors was visualized using BLI imaging of luciferase light production. Tumor-bearing mice treated with vehicle or 40 mg/kg sorafenib were imaged on day 1, 2, 3, and 9. **A:** BLI images of representative mice bearing HCT116-luc2 tumors on day 1, 2, 3 and 9. **B:** Quantitative presentation of the tumor BLI signals (*n* = 5 per group, bar: s.e.m., representative of three independent studies). **C:** Corresponding tumor volume measurements during the imaging study represented as mean and s.e.m. (^#^*p* < 0.05, n = 15 per group, Student’s *t*-test). Results are representative of three independent studies.

**Fig 7:**
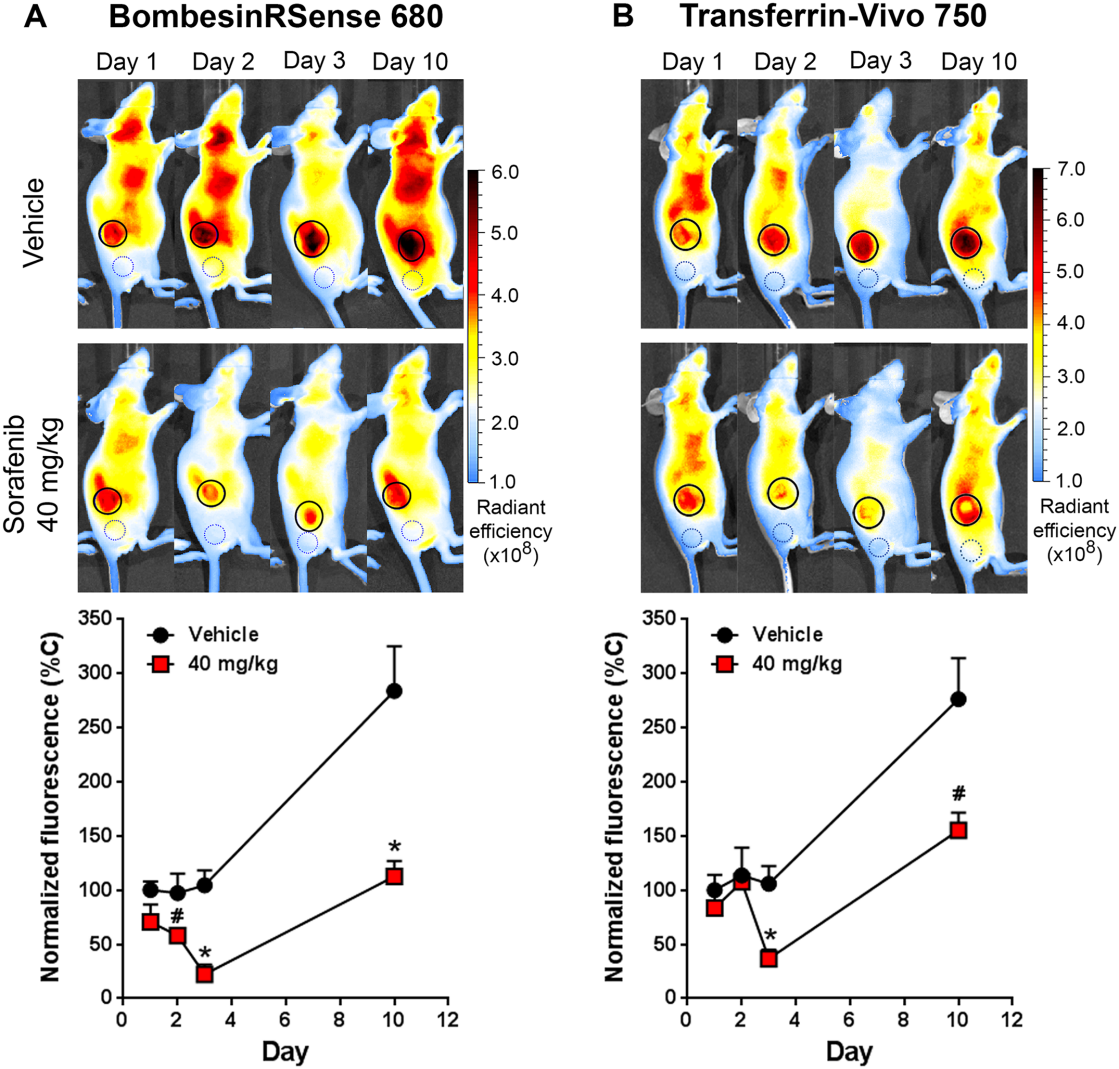
Monitoring subtle tumor metabolic changes in response to low dose sorafenib. Metabolic changes in HCT116-luc2 tumors from the study represented in Fig 6 were visualized using BRS-680 and TfV-750. The probes were injected IV the day before FLI imaging on the IVIS Spectrum CT imaging system. **A:** Representative mouse images are shown for 2D FLI of BRS-680 tumor uptake, with quantitation of tumor fluorescent signal graphed below. Tumor regions of interest (ROI) are represented by solid line circles, and background control ROI are represented by dotted line circles. Average background signal was determined for each probe, normalized for ROI size, and subtracted from each tumor FLI value to more accurately determine levels of inhibition. **B:** Representative mice are shown for TfV-750 uptake in tumors, with quantitation of tumor fluorescent signal graphed below. Results are representative of three independent studies and are shown as mean and s.e.m. (^#^*p* < 0.05, *p < 0.01, day 1–2: *n* = 10, day 3–10: *n* = 5, Student’s *t*-test).

**Fig 8:**
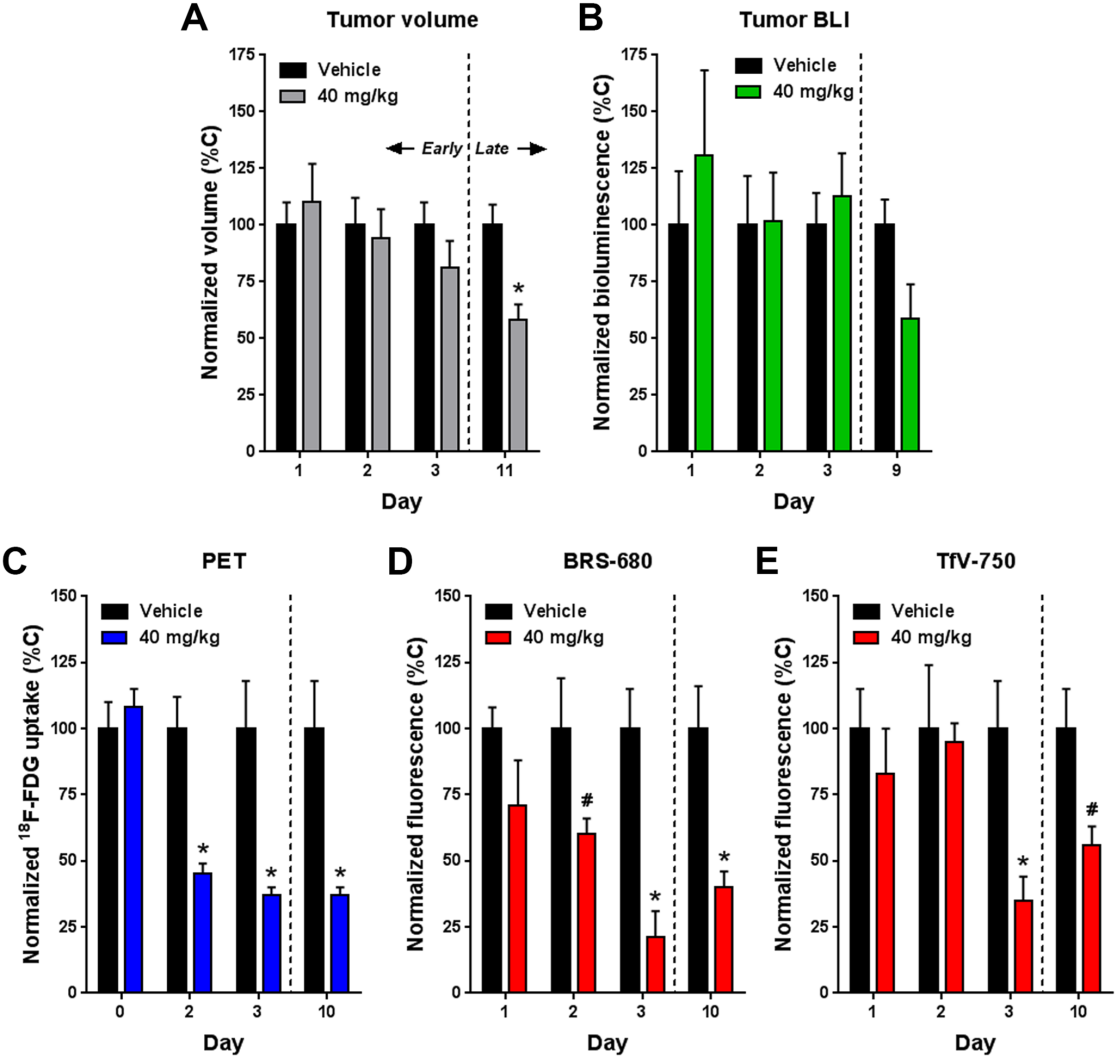
Summary analysis of tumor volume, BLI, PET, and FLI results normalized for comparison. The quantitative imaging data obtained in Figs 4, 6 and 7 were normalized for the mean of each day’s vehicle control group. **A:** Tumor volume (*n* = 15). **B:** BLI signal (*n* = 5). **C:** PET signal (*n* = 5). **D and E:** BRS-680 and TfV-750 FLI signals respectively (day 1–2: *n* = 10, day 3–10: *n* = 5). All data is represented as mean and s.e.m. (#*p* < 0.05, *p < 0.01, Student’s *t*-test). Normalized vehicle group data (means all at 100%) were included to allow representation of error bars for comparison to treatment groups.

Besides interfering with glycolytic activity, we believe inhibition of the Ras/Raf/MEK/ERK pathways with sorafenib has a broader, global impact on tumor energy metabolism. The drug would be expected to affect other metabolism markers such as the bombesin and transferrin receptors, known to be upregulated in a variety of different cancer cells due to their elevated metabolic activities. This means that BRS-680 and TfV-750, effective in imaging terminal outcomes, may also be useful to non-invasively visualize early tumor energy metabolism changes in a manner similar to ^18^F-FDG PET imaging. We wonder if these FLI probes can detect early, yet subtle metabolic disturbances induced by sorafenib treatment prior to the later regression of treated tumors, like we saw with ^18^F-FDG PET imaging. We again used HCT116-luc2 tumor-bearing mice treated with 40 mg/kg of sorafenib, and then compared BLI viability imaging and fluorescent metabolic imaging (BRS-680 and TfV-750) during the first 3 days of treatments. Separate cohorts were used for FLI acquisitions on days 1, 2, and 3 due to the extended time required for probe washout. Of note, the day 1 cohort was re-injected with probe for imaging on day 10 to assess ultimate treatment efficacy. From our previous findings, we know that this drug, at the dose level of 40 mg/kg, does not significantly reduce tumor growth either in terms of tumor bioluminescence or volume (Fig 2B and 2C), and this is seen again in (Fig 6) during the first three days of treatment. However, in the same period of time, our FLI results of BRS-680 (Fig 7A) and TfV-750 (Fig 7B) clearly showed significant reduction in treated tumors as early as day 2 or 3. For better comparisons of different techniques used in this study, we normalized our imaging data from Figs 4, 6 and 7 to the vehicle control animals (as 100%) and summarized the findings in Fig 8. Conventional tumor volume measurement (Fig 8A) and BLI imaging (Fig 8B) were not able to detect early change in tumors (Fig 8A and 8B) when compared with the gold standard PET imaging (Fig 8C), On the other hand, the BRS-680 FLI imaging indeed detected early drop in energy metabolism in a fashion similar to the PET imaging (Fig 8D). Although less sensitive than BRS-680, TfV-750 indeed detected reduced tumor metabolic activity as early as 3 days post treatment (Fig 8E).

## Discussion

Cancer cells typically acquire several mutations in pivotal pro-oncogenic signaling regulators in order to cope with their survival needs. These regulators serve as master switches controlling major cellular functions such as energy metabolism, proliferation, angiogenesis and metastasis. In light of their oncogenic properties, these regulators have become the key targets for anti-cancer drug development. Many specific inhibitors have been developed against these targets in order to achieve specific killing of cancer cells. It is expected that, during the course of a targeted therapy, a tumor would experience several phases of biological changes before its death (Fig 3), including alterations in metabolism [35], proliferation [36], angiogenesis [37], and inflammation [38]. Among them, a reduction in tumor metabolic activity should be the foremost and most impactful event immediately following treatment, since cell growth and division involves many energy demanding biochemical processes (e.g. DNA, RNA, and protein biosynthesis, and mitosis). Thus, an easy and robust molecular imaging method capable of detecting tumor metabolic changes would be of great interest for development of next generation anti-cancer drugs. In that regard, drugs with specific effects on tumor energy metabolism at a lower dose may provide better therapeutic outcome when administrated at a higher dose or in prolonged treatment.

In the report, we demonstrated the collaborative use of multimodal imaging, including BLI, FLI and PET, for a more comprehensive picture of tumor’s metabolic status, especially during the early phase of targeted therapy. Each modality looks at a different aspect of cancer biology. To date, PET is the most frequently used molecular imaging method in the clinic for imaging glucose metabolism in tumors. As cancer cells have abnormally higher glycolysis activities than normal cells, ^18^F-FDG PET imaging has been a standard and conventional approach for cancer imaging in patients [4]. This imaging tool is not only used for cancer detection and diagnosis, but also used for its capability to visualize early responsiveness in tumors to targeted therapy [4]. For example, in patients with gastrointestinal stromal tumor (GIST), ^18^F-FDG PET imaging has been used to detect early reduction in glucose uptake in tumor after the imatinib treatment. In a similar pre-clinical GIST animal study, ^18^F-FDG PET also detected an early response to nilotinib treatment [17].

However, as PET has a relatively low throughput and involves the use of radioactive tracers, it’s adaptation in pre-clinical settings is rather limited. Many optical imaging systems, such as the IVIS optical imaging platform used in the study, are capable of imaging of multiple subjects simultaneously, while PET only images one subject at a time in most instrumental settings. In particular, FLI is particularly suitable for *in vivo* studies that involve screening a large volume of chemical entities in order to identify lead candidates for later development. Using FLI techniques to directly visualize glucose uptake has also been reported. A green fluorescent glucose analog 2-NBDG has been synthesized for microscopic imaging of glucose uptake in cancer cells [39]. However, as green light has limited tissue penetration, 2-NBDG is not ideal for non-invasive imaging of glucose uptake in living animals. Putative glucose probes that emit fluorescence at more suitable NIR wavelengths, due to their large size, do not show performance consistent with actual cellular uptake as is seen with ^18^F-FDG [17]. Thus, in this study, we demonstrated an alternative approach and used other types of NIR FLI probes to monitor early tumor metabolic responses to a targeted therapeutics. Instead of targeting glucose uptake mechanism, this type of probe targets important surface biomarkers that are closely correlated with energy metabolism status in cancer cells. More importantly, this non-radioactive imaging strategy has the advantage over PET for its capability of simultaneous imaging of multiple biomarkers using probes with different spectral properties. If needed, more than two fluorescent probes can be used and each fluorescent channel can be individually identified using the spectral unmixing feature of our LivingImage^®^ software. PET imaging is intrinsically limited in this regard, as all PET isotopes emit the same positron that is not distinguishable by the detector.

In addition to FLI, we used the IVIS imaging platform to acquire BLI images for tumor viability assessment. The platform’s BLI technology has been widely adopted for pre-clinical imaging of small animal tumor xenograft models. To generate BLI signals, human cancer cells are stably transfected with a firefly luciferase expression cassette prior to inoculation into immunocompromised rodents to establish tumor growth. Equipped with a highly sensitive camera, IVIS BLI has excellent sensitivity and specificity. One of the greatest advantage of BLI is its superior signal-to-noise ratio; the firefly luciferase expression is limited to tumor cells but not present anywhere else in normal tissues. In this study, we used BLI to identify the minimal dosage of sorafenib that suppresses but does not sufficient for complete eradication. The enzyme’s light producing reaction involves oxidation of its specific substrate D-luciferin in the presence of molecular oxygen and ATP [40]. The need of ATP for light generation makes BLI a useful indicator for assessing tumor viability in response to different anti-cancer treatments, especially when conventional tumor volume measurement fails to indicate any difference.

As each imaging modality looks at different perspective of cancer biology, the collaborative use of BLI, FLI and PET imaging provides a more comprehensive view of the tumor. This could potentially expand our ability to simultaneously assess a variety of biological and physiological events in tumors, not only limited to metabolic imaging. For example, normalization of our tumor volume, BLI, PET and FLI results at each time point allows us to best highlight treatment effects and the similarities and differences between the different readouts (Fig 8). In this case, we were interested in benchmarking our optical imaging to the gold standard of ^18^F-FDG PET imaging. Tumor volume (Fig 8A) and BLI (Fig 8B) measurements did not show definitive sorafenib anti-tumor effects until days 9–11. In contrast, PET imaging (Fig 8C) could detect early treatment effects (50–60%) on tumor metabolism on days 2 and 3, when tumor volumes were not affected. PET imaging results also showed great treatment responsiveness in the later phase on day 10, however the decreased ^18^F-FDG uptake would be due to a combination of altered tumor metabolism as well as decreased tumor burden. It is interesting to see that BRS-680 imaging (Fig 8D) could also detect 40–80% inhibition of tumor signal by sorafenib treatment within the 2–3 day window in which tumor volume is unaffected, for the first time showing that NIR FLI has the potential to provide data similar to ^18^F-FDG PET imaging. TfV-750 imaging could also detect early sorafenib effects on metabolism (~70% inhibition), but only as early as day 3, suggesting that metabolic changes in transferrin receptor expression are a little slower to be affected by this treatment. Both FLI probes, as in Fig 5, were effective in detecting treatment effects on day 10 (Fig 8D and 8E).

In summary, we have demonstrated the synergistic use of multimodal imaging to depict a comprehensive picture of the tumor response to drug treatment. Combining the PET, BLI and FLI imaging technologies, we were able to detect early changes in response to targeted cancer therapy prior to changes in viability status. More importantly, our results show that disturbances in metabolic activity occurred very early, within 2–3 days of treatment, and our FLI approach was corroborated by the gold standard ^18^F-FDG PET in metabolic imaging. We believe this optical imaging approach may be a useful alternative for pre-clinical development of anti-cancer drugs that are designed to target tumor energy metabolism.

## Supporting information

S1 Fig Fluorescent spectral property of BRS-680. Probe spectral analysis was assessed by spectrophotometer and indicates that BRS-680 has maximum absorption at 674 nm, and maximum emission at 690 nm.
(TIF)

S2 Fig BRS-680 is a selective imaging probe for bombesin receptor. BRS-680 was generated from a 7 amino acid peptide from gastrin-releasing peptide (GRP) that was used as a targeting moiety for the bombesin receptor. **A:** Fluorescence microscopy shows BRS-680 (1 μM) binding to cultured HT-29 human colorectal cells and competitive blockade by pre-incubation with unlabeled 100 μM native GRP peptide. **B:** Flow cytometry analysis of cells labeled with BRS-680, scramble peptide control, or in the presence of unlabeled 100X GRP peptide. **C:** Fluorescence microscopy of excised tumor section labeled with 1 μM BRS-680 in the presence or absence of excess GRP peptide (100 μM) to compete for binding.
(TIF)

S3 Fig Pharmacokinetics and biodistribution of BRS-680 in mice. **A:** CD-1 mice were injected IV with 2 nmoles of BRS-680. Blood samples were obtained via terminal cardiac puncture and plasma fluorescence was quantified on a fluorescence plate reader. The circulation half-life was measured as ~1.7h. **B:** HT-29 tumor-bearing mice received an IV injection of 2 nmoles BRS-680 in a final volume of 100 μl. Mice were sacrificed 24 hours post-injection and tissues were excised, rinsed with PBS, and imaged on the FMT 4000 (PerkinElmer, Waltham, MA), in epifluorescence mode. Epifluorescence levels were determined using TrueQuant^™^ FMT software and are represented as Mean Fluorescence to compensate for differences in tissue size.
(TIF)

S4 Fig Fluorescence kinetics of BRS-680 in tumor-bearing mice. HT-29 tumor-bearing mice (tumors on both left and right flanks) were injected with 2 nmoles BRS-680 and imaged on the FMT 4000 at multiple time points 6 to 144 h later. Noninvasive fluorescence tomographic imaging datasets were used to quantify kinetic fluorescence changes in the tumors, heart, and liver. An optimal imaging time point of 24h was determined based on specificity of tumor signal and tumor’s signal-to-background ratio.
(TIF)

S5 Fig Binding of BRS-680 co-localizes with the BB_2_R bombesin receptor on excised tumor section. To confirm specific tumor localization of BRS-680 *in vivo* (S4 Fig), tumors were excised at 24h, and BRS-680 fluorescence (red) in 10 μm sections, was compared to adjacent serial sections stained with a rabbit polyclonal antibody against GRP receptor (BB_2_R, green). Sections were imaged by fluorescence microscopy using DAPI (blue) as a nuclear counterstain.
(TIF)

S6 Fig Fluorescent spectral property of TfV-750. Spectral analysis assessed by spectrophotometer indicates that BRS-680 has maximum absorption at 750 nm, and maximum emission at 770 nm.
(TIF)

S7 Fig Specific binding of TfV-750 to transferrin receptor on cancer cells. **A:** HT-29 cells (human colon cancer tumor line) were incubated with 1 μM TfV-750 for 1 h at room temperature and assessed by flow cytometry, showing effective uptake in the tumor cells. **B:** Titration of TfV-750 showed the dose dependence of cell uptake. Of note, prior incubation with 100 μM unlabeled transferrin blocked uptake of 1 μM TfV-750. **C:** Generation of cellular microscopy images required the use of a 645 nm fluorochrome-labeled transferrin to more sensitively detect cellular fluorescence by microscopy. Transferrin 645 was incubated with cells for 1h at room temperature under the conditions described in B, with and without blockade by excess unlabeled transferrin.
(TIF)

S8 Fig Pharmacokinetics and biodistribution of TfV-750 in mice. TfV-750 was injected IV (2 nmol/mouse) and tissues were collected 24 h later for biodistribution assessment. **A:** Epifluorescence images of excised tissues shows predominant tumor, liver, and kidney signal. **B:** Quantification of tissue epifluorescence images, with data inset representing plasma pharmacokinetics assessed from serial bleeds of mice measured on a microplate fluorimeter.
(TIF)

S9 Fig Fluorescent kinetics property of TfV-750 in tumor-bearing mice. HT-29 tumor bearing mice were imaged at different time points after TfV-750 injection (2 nmol/mouse). Elevated TfV-750 signals in the liver and tumor indicate higher iron metabolism in these tissues.
(TIF)

S10 Fig Fluorescently labeled transferrin specifically targets tumors. Specificity of tumor localization of transferrin receptor targeting was determined by IV injection of mice with 645 nm fluorochrome-labeled Transferrin (as a surrogate for TfV-750) that allows sensitive detection by fluorescence microscopy. In addition, Hoechst 33342 was injected 5 minutes prior to animal termination (to label vasculature regions). Tissues from injected, tumor-bearing mice were excised and flash frozen prior to sectioning (10 μm in thickness). The tissue sections were then stained with FITC-labeled anti-transferrin receptor (TfR) antibody. The acquired images confirm non-colocalization with the vasculature and good co-localization with TfR staining.
(TIF)

S11 Fig The ARRIVE Guideline checklist. The checklist contains information to improve experimental reporting of animal studies for purpose of post-publication data analysis and reproducibility.
(DOCX)
