# Supplementary figures and images for: Fluorescence imaging of bombesin and transferrin receptor expression is comparable to 18F-FDG PET in early detection of sorafenib-induced changes in tumor metabolism
Source: PLoS One. 2017 Aug 8;12(8):e0182689. doi: 10.1371/journal.pone.0182689 (PMC5549732; doi:10.1371/journal.pone.0182689)

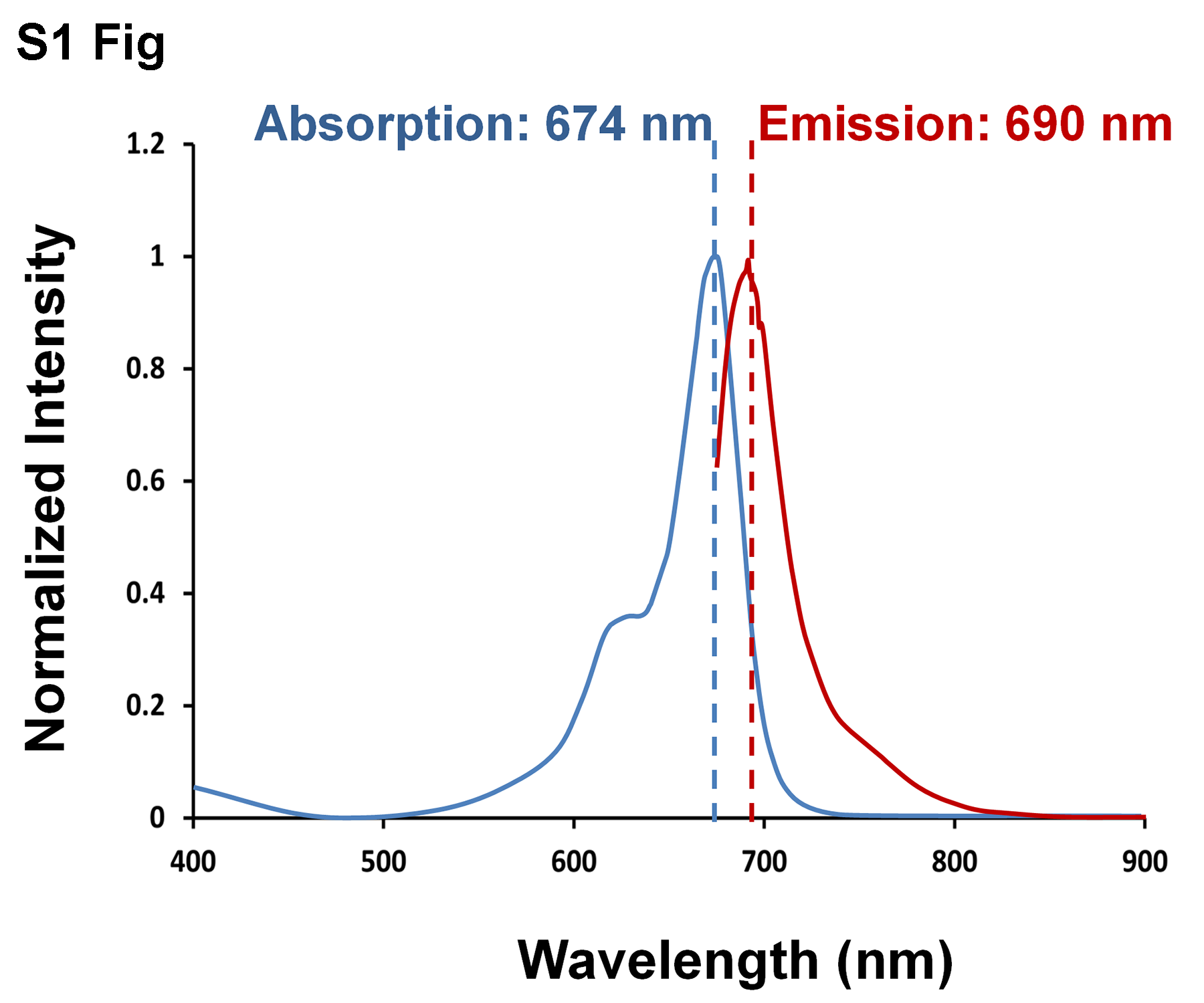

Supplement: S1 Fig — Probe spectral analysis was assessed by spectrophotometer and indicates that BRS-680 has maximum absorption at 674 nm, and maximum emission at 690 nm. (TIF) [file pone.0182689.s001.tif]

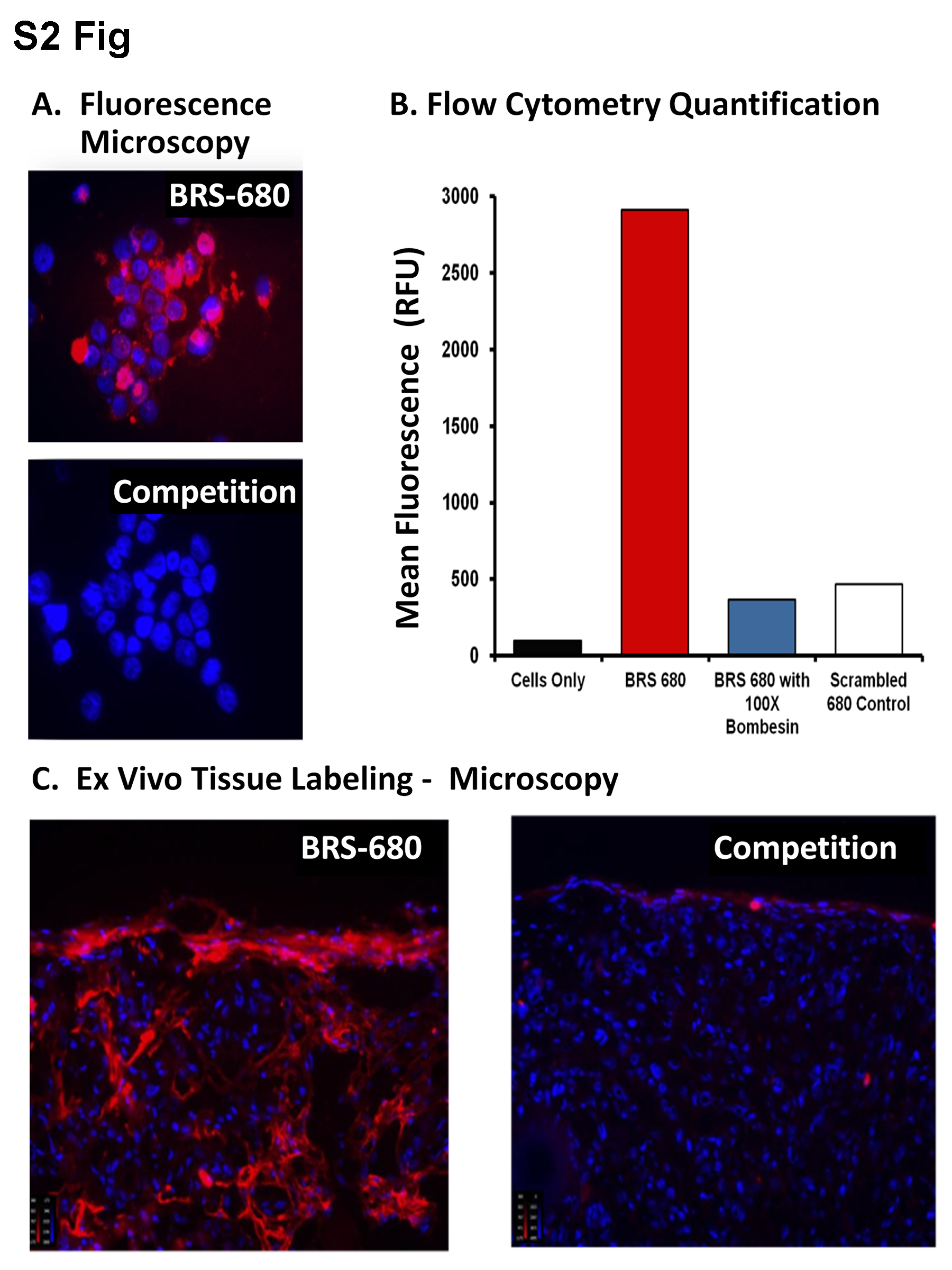

Supplement: S2 Fig — BRS-680 was generated from a 7 amino acid peptide from gastrin-releasing peptide (GRP) that was used as a targeting moiety for the bombesin receptor. A: Fluorescence microscopy shows BRS-680 (1 μM) binding to cultured HT-29 human colorectal cells and competitive blockade by pre-incubation with unlabeled 100 μM native GRP peptide. B: Flow cytometry analysis of cells labeled with BRS-680, scramble peptide control, or in the presence of unlabeled 100X GRP peptide. C: Fluorescence microscopy of excised tumor section labeled with 1 μM BRS-680 in the presence or absence of excess GRP peptide (100 μM) to compete for binding. (TIF) [file pone.0182689.s002.tif]

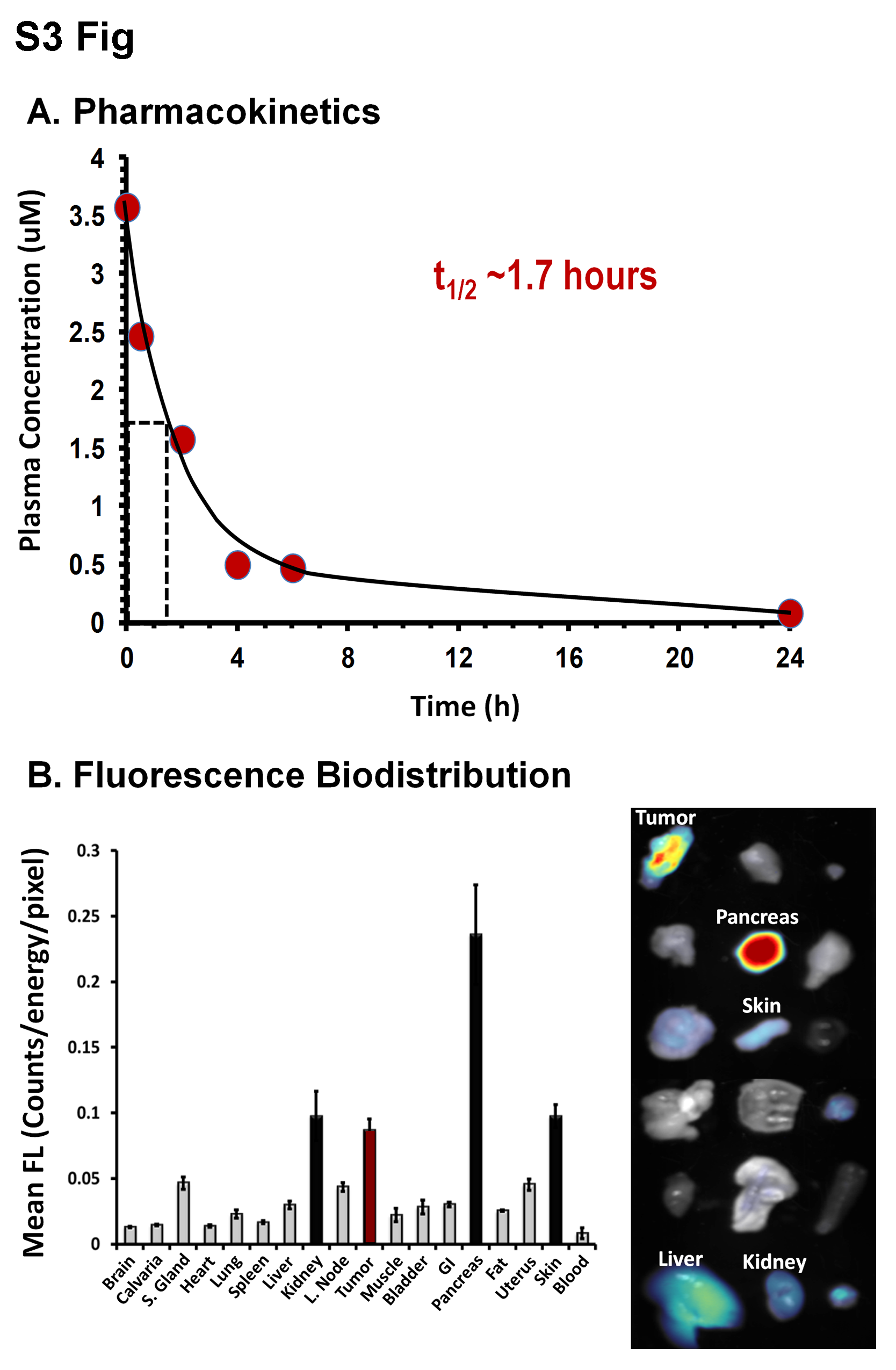

Supplement: S3 Fig — A: CD-1 mice were injected IV with 2 nmoles of BRS-680. Blood samples were obtained via terminal cardiac puncture and plasma fluorescence was quantified on a fluorescence plate reader. The circulation half-life was measured as ~1.7h. B: HT-29 tumor-bearing mice received an IV injection of 2 nmoles BRS-680 in a final volume of 100 μl. Mice were sacrificed 24 hours post-injection and tissues were excised, rinsed with PBS, and imaged on the FMT 4000 (PerkinElmer, Waltham, MA), in epifluorescence mode. Epifluorescence levels were determined using TrueQuant™ FMT software and are represented as Mean Fluorescence to compensate for differences in tissue size. (TIF) [file pone.0182689.s003.tif]

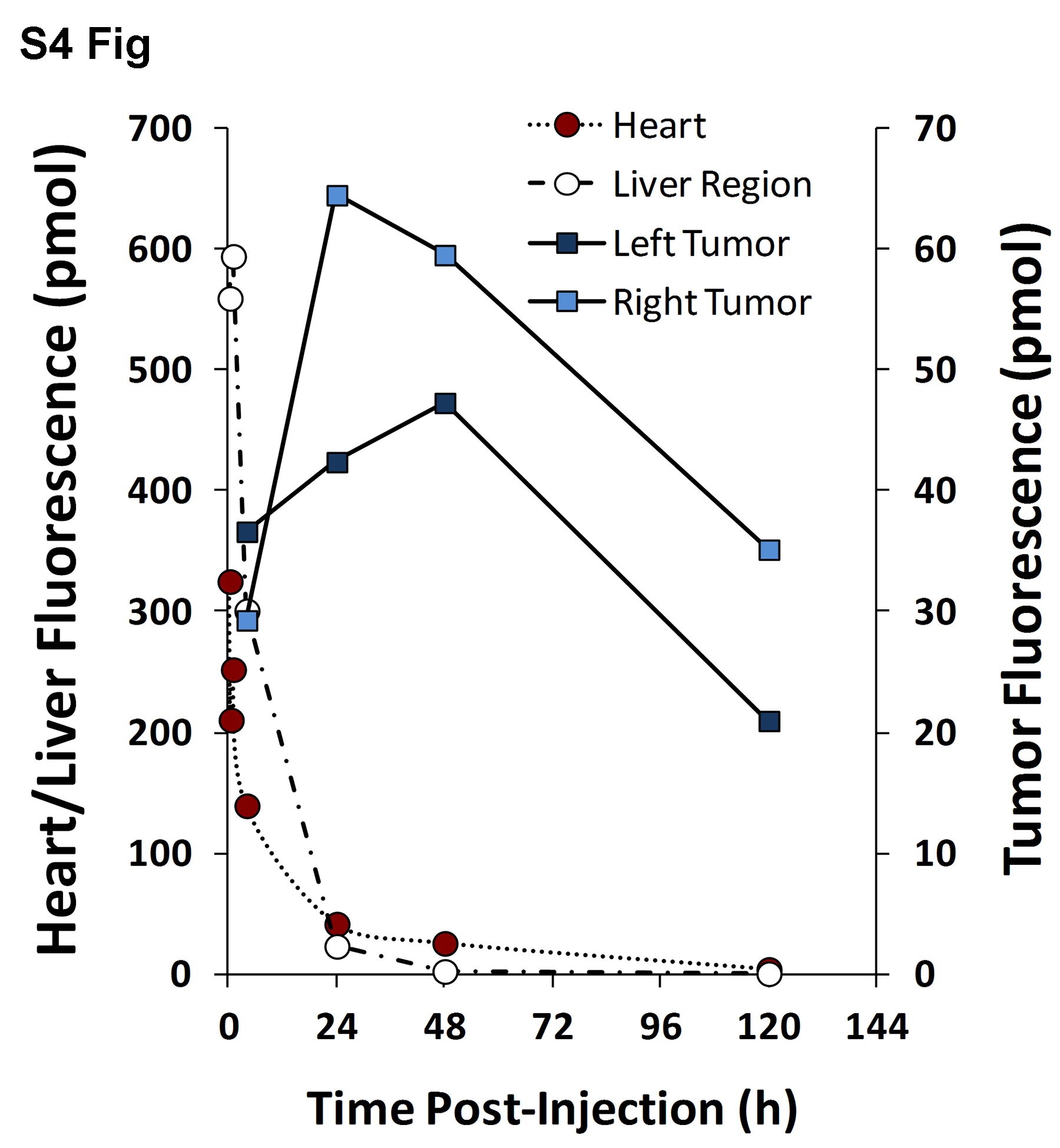

Supplement: S4 Fig — HT-29 tumor-bearing mice (tumors on both left and right flanks) were injected with 2 nmoles BRS-680 and imaged on the FMT 4000 at multiple time points 6 to 144 h later. Noninvasive fluorescence tomographic imaging datasets were used to quantify kinetic fluorescence changes in the tumors, heart, and liver. An optimal imaging time point of 24h was determined based on specificity of tumor signal and tumor’s signal-to-background ratio. (TIF) [file pone.0182689.s004.tif]

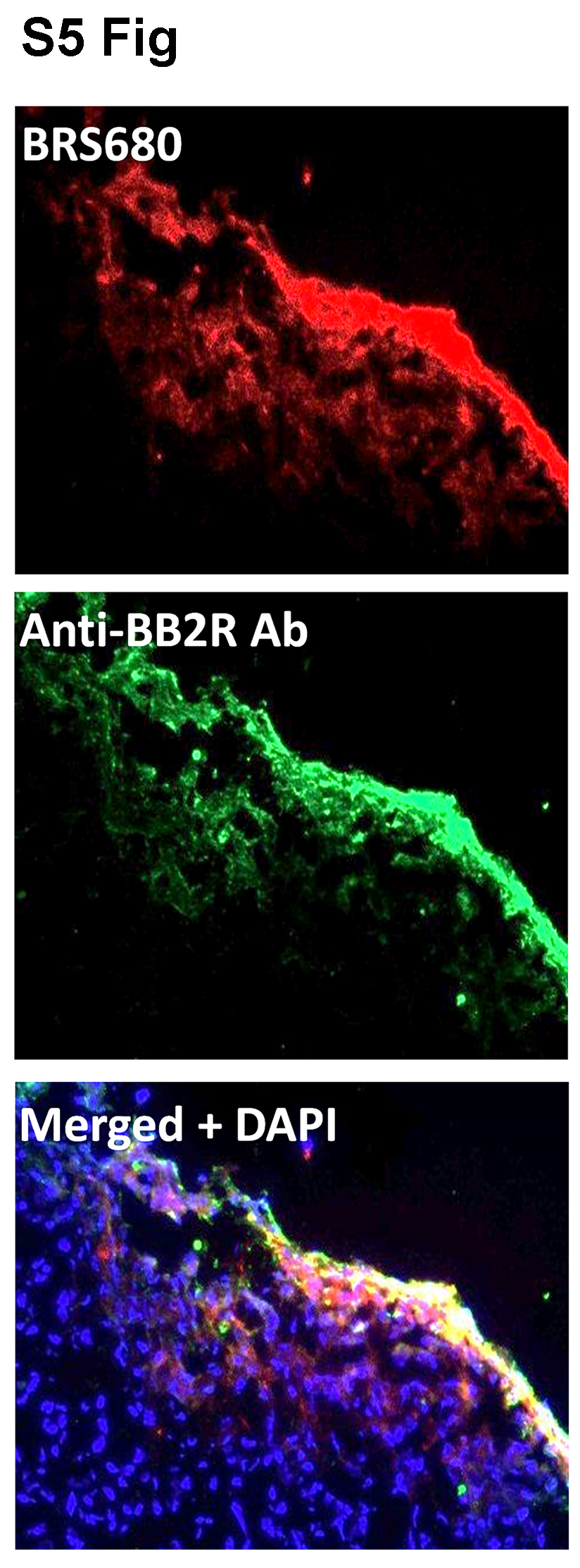

Supplement: S5 Fig — To confirm specific tumor localization of BRS-680 in vivo (S4 Fig), tumors were excised at 24h, and BRS-680 fluorescence (red) in 10 μm sections, was compared to adjacent serial sections stained with a rabbit polyclonal antibody against GRP receptor (BB2R, green). Sections were imaged by fluorescence microscopy using DAPI (blue) as a nuclear counterstain. (TIF) [file pone.0182689.s005.tif]

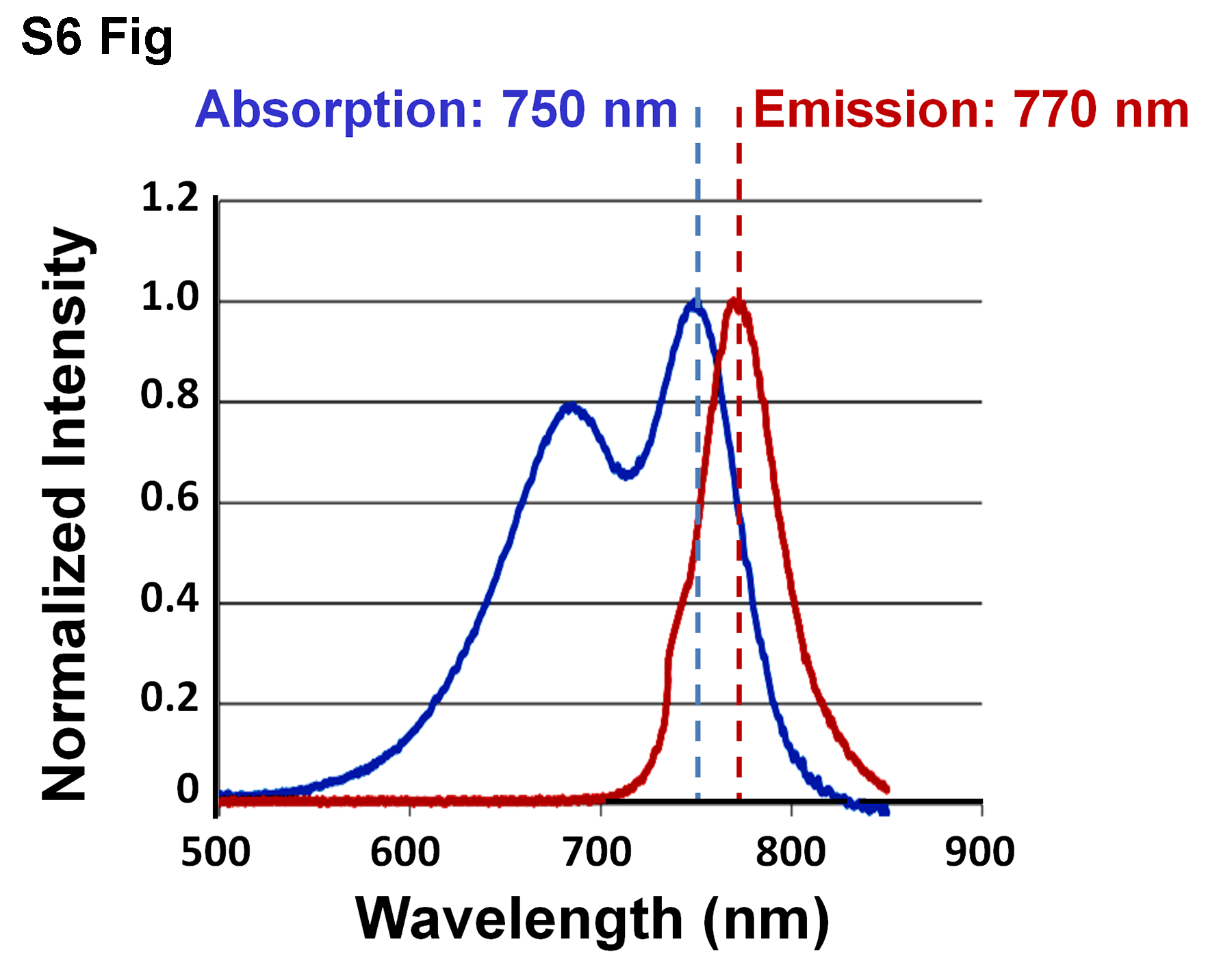

Supplement: S6 Fig — Spectral analysis assessed by spectrophotometer indicates that BRS-680 has maximum absorption at 750 nm, and maximum emission at 770 nm. (TIF) [file pone.0182689.s006.tif]

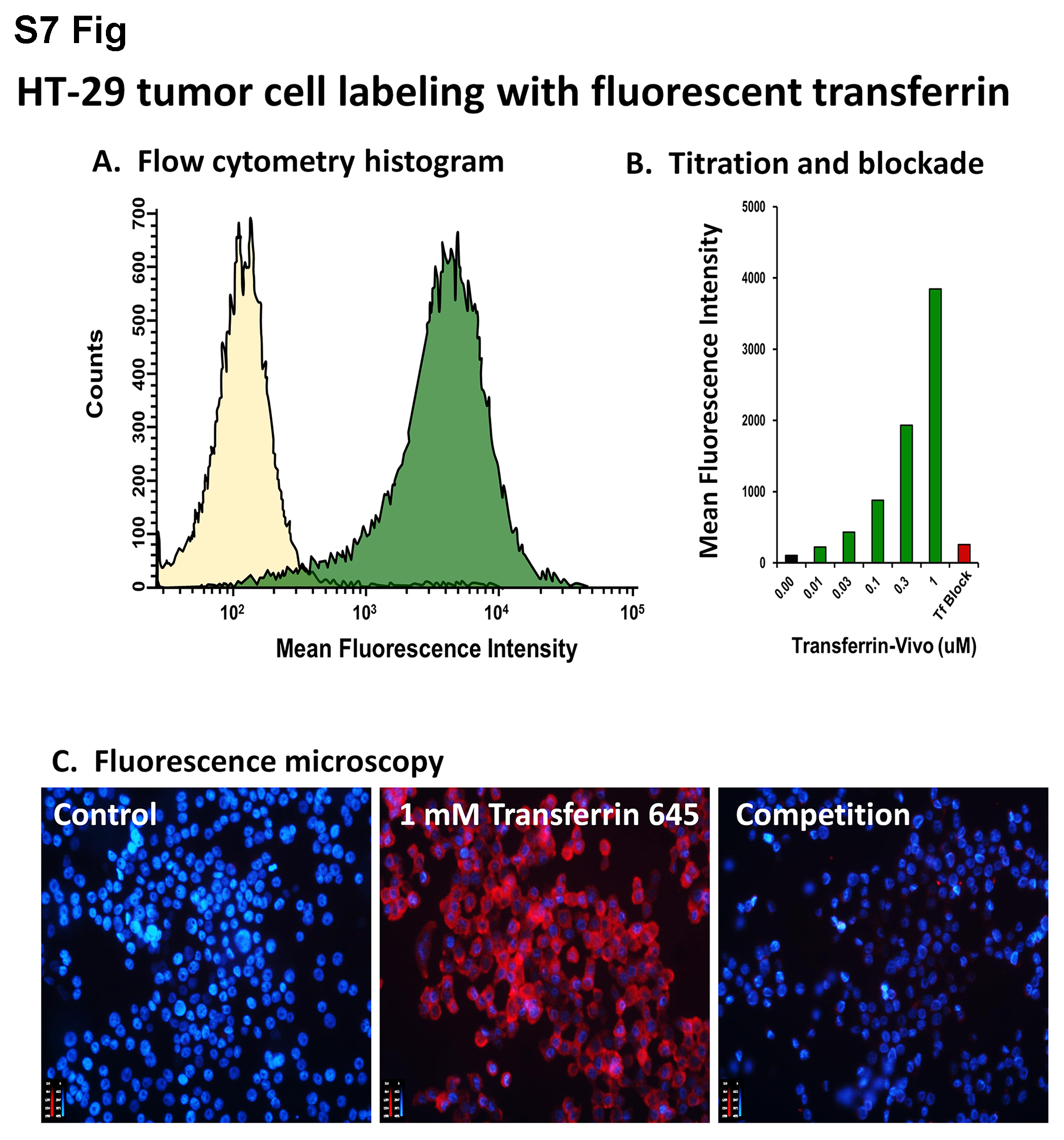

Supplement: S7 Fig — A: HT-29 cells (human colon cancer tumor line) were incubated with 1 μM TfV-750 for 1 h at room temperature and assessed by flow cytometry, showing effective uptake in the tumor cells. B: Titration of TfV-750 showed the dose dependence of cell uptake. Of note, prior incubation with 100 μM unlabeled transferrin blocked uptake of 1 μM TfV-750. C: Generation of cellular microscopy images required the use of a 645 nm fluorochrome-labeled transferrin to more sensitively detect cellular fluorescence by microscopy. Transferrin 645 was incubated with cells for 1h at room temperature under the conditions described in B, with and without blockade by excess unlabeled transferrin. (TIF) [file pone.0182689.s007.tif]

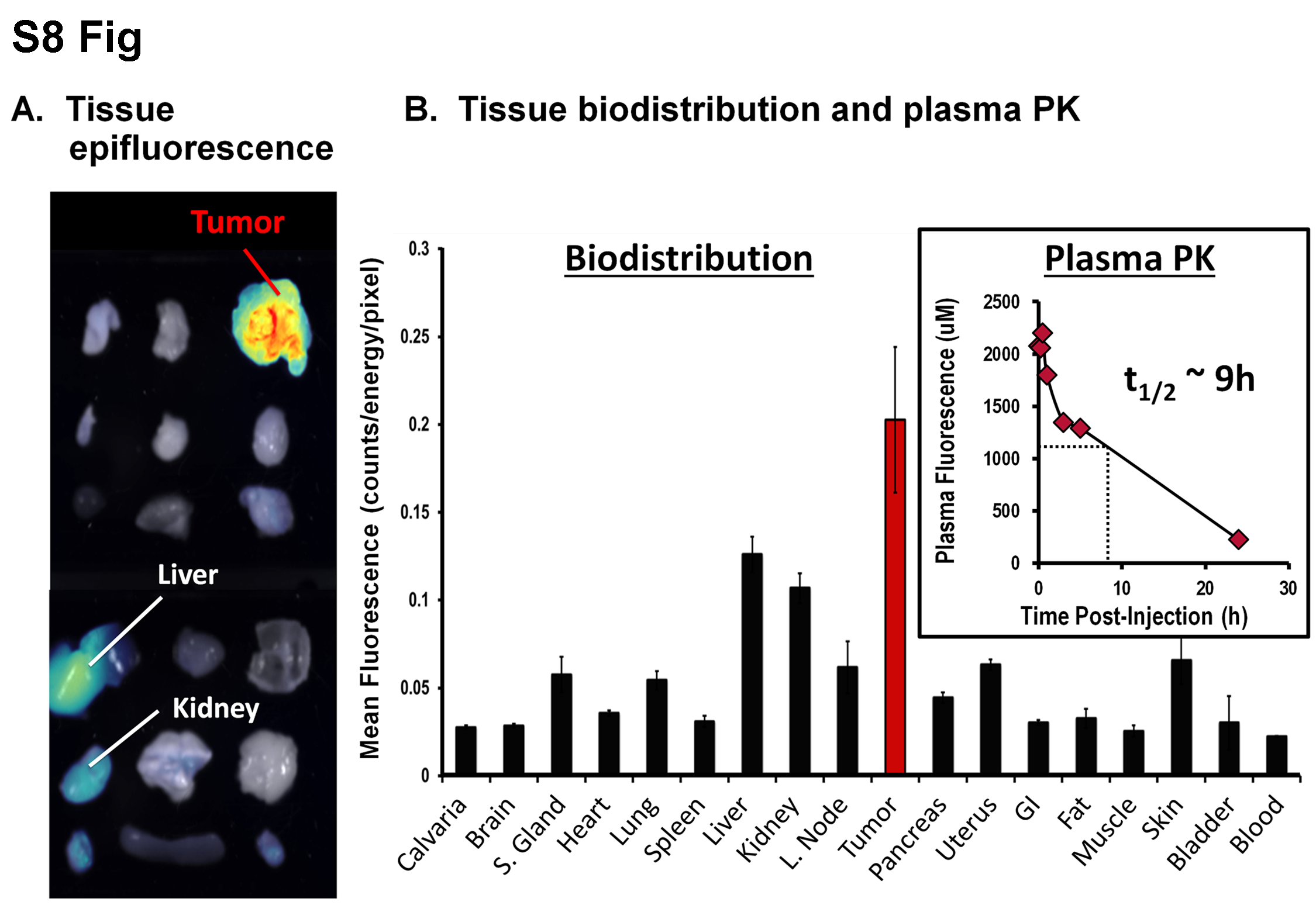

Supplement: S8 Fig — TfV-750 was injected IV (2 nmol/mouse) and tissues were collected 24 h later for biodistribution assessment. A: Epifluorescence images of excised tissues shows predominant tumor, liver, and kidney signal. B: Quantification of tissue epifluorescence images, with data inset representing plasma pharmacokinetics assessed from serial bleeds of mice measured on a microplate fluorimeter. (TIF) [file pone.0182689.s008.tif]

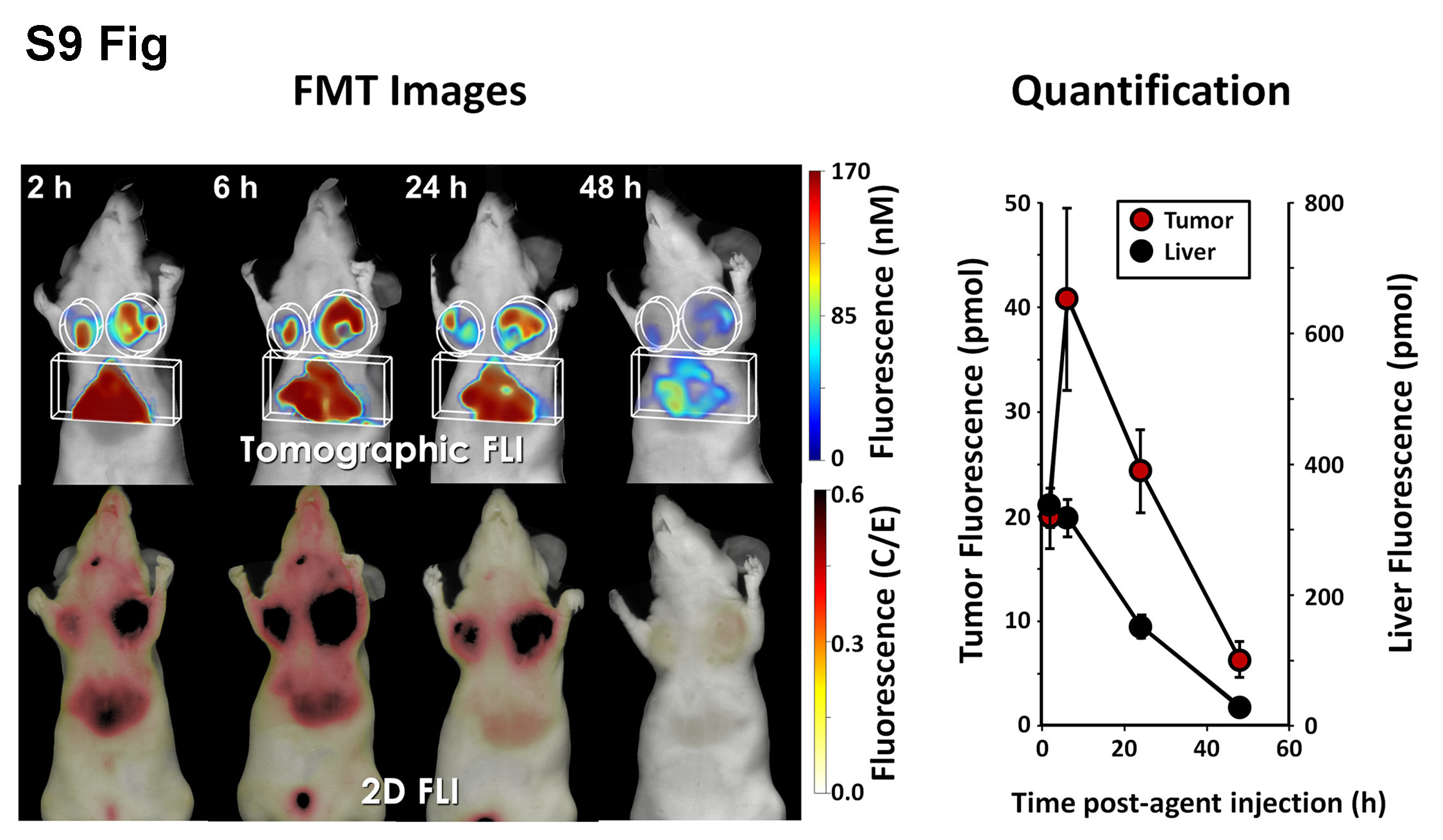

Supplement: S9 Fig — HT-29 tumor bearing mice were imaged at different time points after TfV-750 injection (2 nmol/mouse). Elevated TfV-750 signals in the liver and tumor indicate higher iron metabolism in these tissues. (TIF) [file pone.0182689.s009.tif]

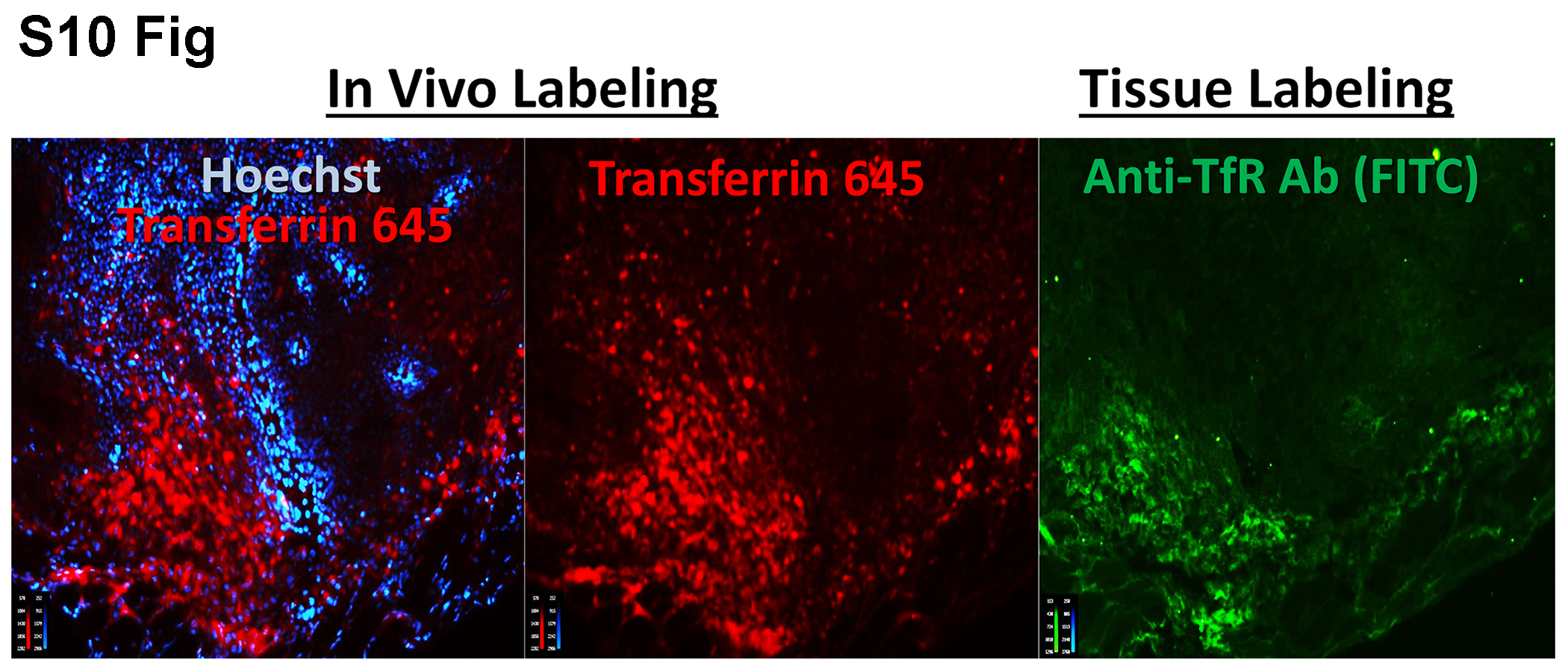

Supplement: S10 Fig — Specificity of tumor localization of transferrin receptor targeting was determined by IV injection of mice with 645 nm fluorochrome-labeled Transferrin (as a surrogate for TfV-750) that allows sensitive detection by fluorescence microscopy. In addition, Hoechst 33342 was injected 5 minutes prior to animal termination (to label vasculature regions). Tissues from injected, tumor-bearing mice were excised and flash frozen prior to sectioning (10 μm in thickness). The tissue sections were then stained with FITC-labeled anti-transferrin receptor (TfR) antibody. The acquired images confirm non-colocalization with the vasculature and good co-localization with TfR staining. (TIF) [file pone.0182689.s010.tif]
