# Supplementary material for: Fluorescence imaging of bombesin and transferrin receptor expression is comparable to 18F-FDG PET in early detection of sorafenib-induced changes in tumor metabolism
Source: PLoS One. 2017 Aug 8;12(8):e0182689. doi: 10.1371/journal.pone.0182689 (PMC5549732; doi:10.1371/journal.pone.0182689)

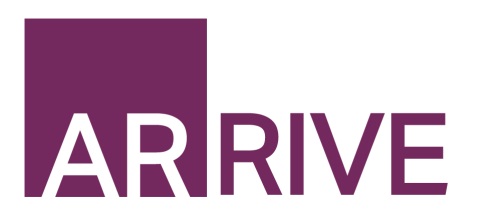


The ARRIVE Guidelines Checklist

Animal Research: Reporting In Vivo Experiments

Carol Kilkenny^1^, William J Browne^2^, Innes C Cuthill^3^, Michael Emerson^4^ and Douglas G Altman^5^

*^1^The National Centre for the Replacement, Refinement and Reduction of Animals in Research, London, UK, ^2^School of Veterinary Science, University of Bristol, Bristol, UK, ^3^School of Biological Sciences, University of Bristol, Bristol, UK, ^4^National Heart and Lung Institute, Imperial College London, UK, ^5^Centre for Statistics in Medicine, University of Oxford, Oxford, UK.*

|  | | ITEM | RECOMMENDATION | Section/ Paragraph |
| --- | --- | --- | --- | --- |
| 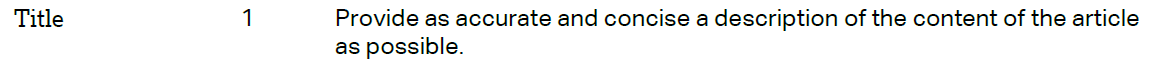 | | | Title |  |
| 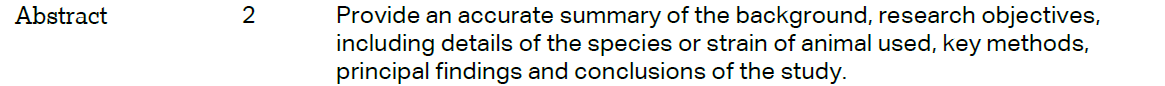 | | | Abstract |  |
| INTRODUCTION | | |  |  |
| 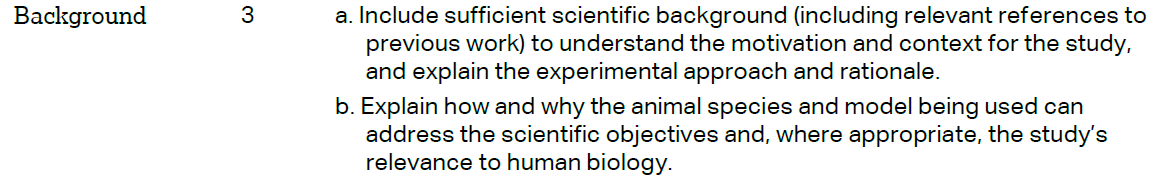 | | | Paragraphs 1-4  Paragraphs 3-5 |  |
| 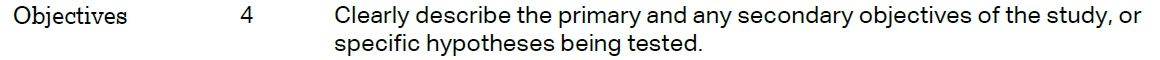 | | | Paragraph 5 |  |
| METHODS | | |  |  |
| 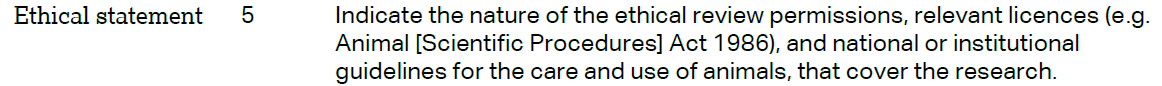 | | | Paragraph 1 |  |
| 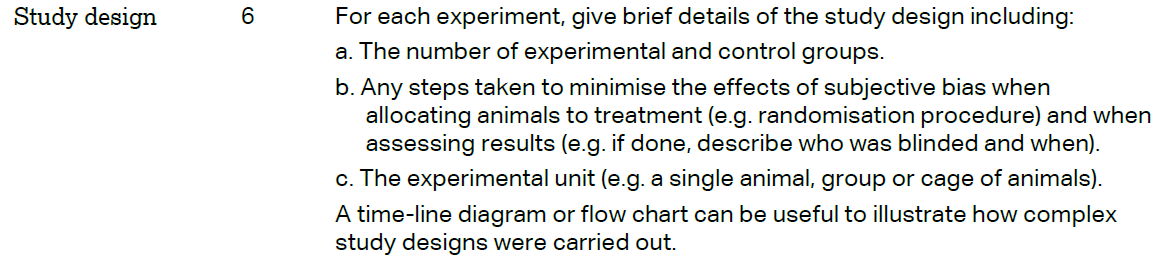 | | | Paragraphs 6-8  Paragraph 9  Paragraph 7-9  Figure 3 |  |
| 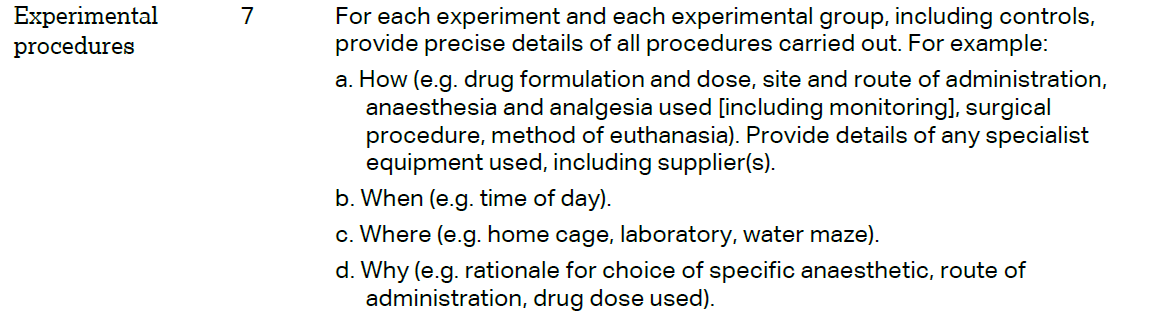 | | | Paragraphs 3, 6-8 |  |
| 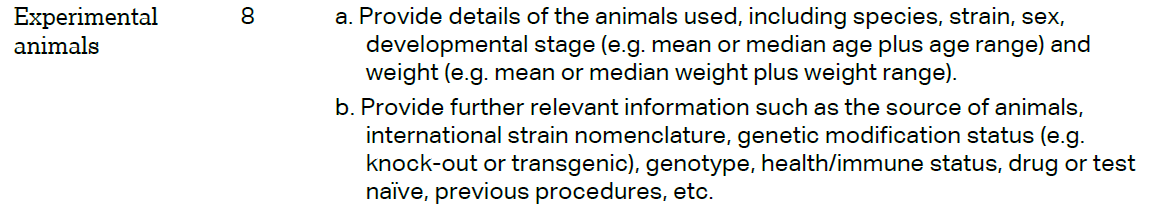 | | | Paragraph 3 |  |

The ARRIVE guidelines. Originally published in *PLoS Biology*, June 2010^1^

| 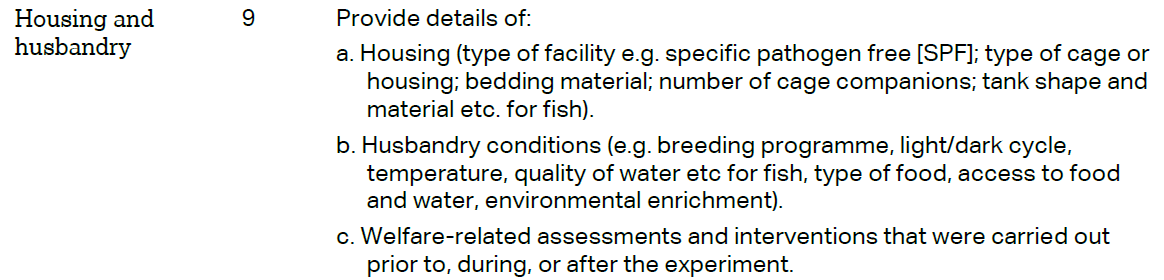 | Paragraph 3 | |
| --- | --- | --- |
| 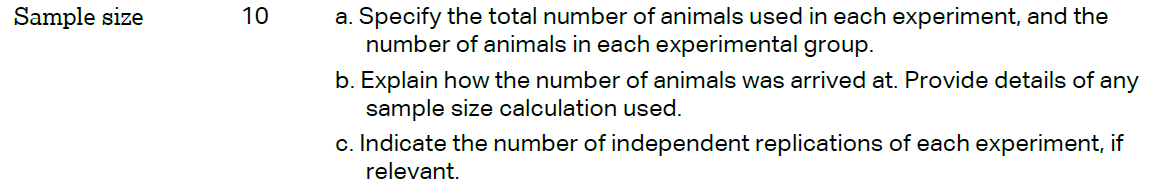 | Paragraphs 6-8  Paragraph 9  Figure legends 1-8 | |
| 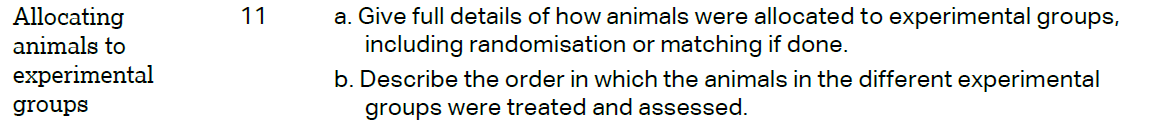 | Paragraph 3  Paragraphs 6-8 | |
| 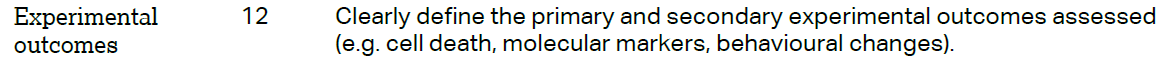 | Paragraphs 3, 6-8 | |
| 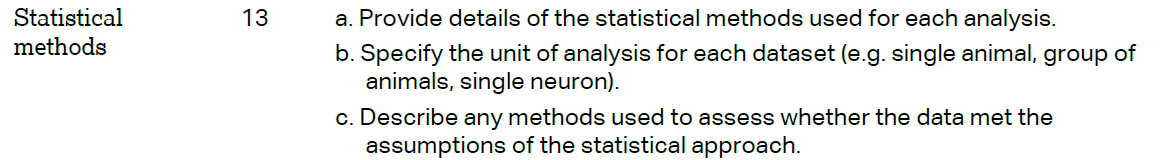 | Paragraph 9  Paragraph3, 6-8 | |
| RESULTS |  | |
| 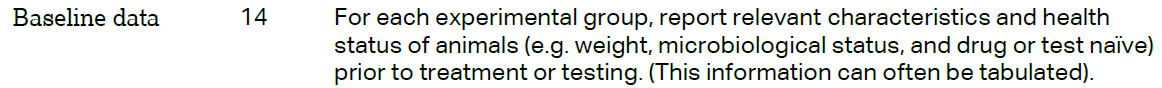 | Method paragraph 3 | |
| 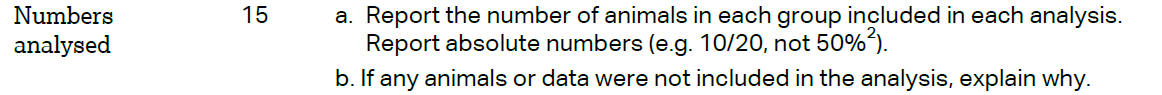 | Figure legends 1-2, 4-8 | |
| 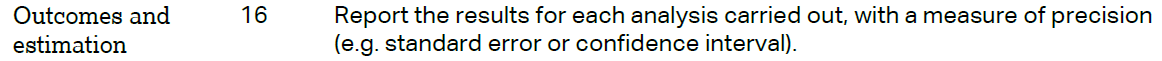 | Figures 1-2, 4-8 | |
| 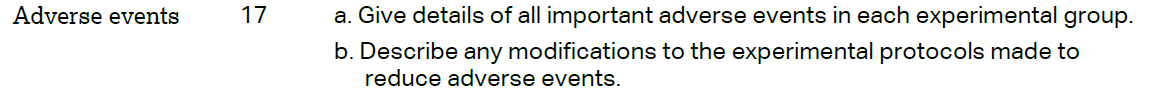 | Paragraph 3  Figure 2 | |
| DISCUSSION |  | |
| 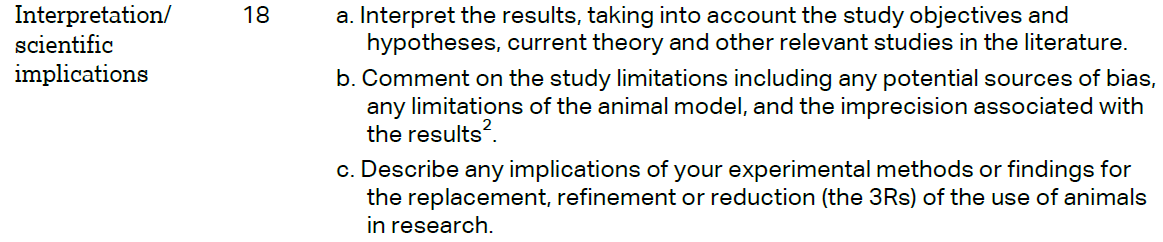 | Paragraphs 2-5  Paragraph 5  Paragraph 7 | |
| 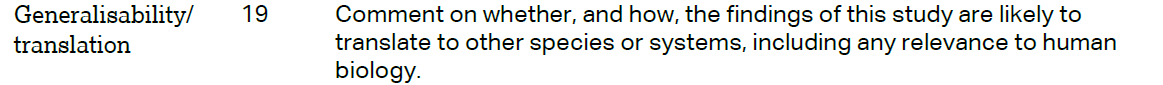 | Paragraphs 2-5 | |
| 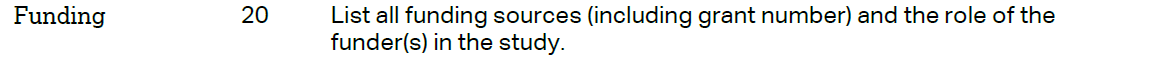 | | Acknowledgements |


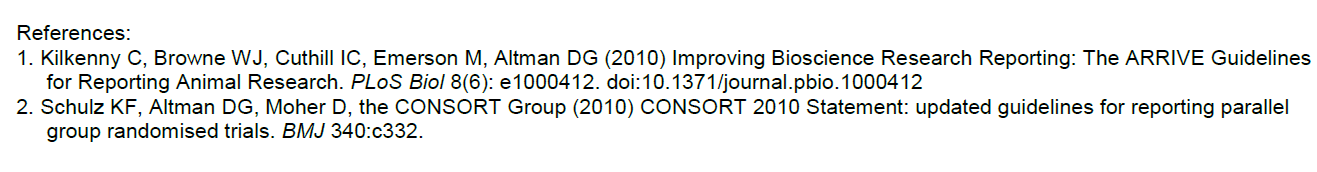

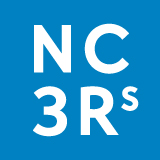

Supplement: S11 Fig — The checklist contains information to improve experimental reporting of animal studies for purpose of post-publication data analysis and reproducibility. (DOCX) [file pone.0182689.s011.docx]
